# Cryopreserved umbilical allograft (equicenta® CTM) improves clinical outcomes compared with clinician-selected standard of care in performance horses: a pragmatic prospective controlled trial

**DOI:** 10.3389/fvets.2026.1833933

**Published:** 2026-08-04

**Authors:** Alicia L. Bertone, Alec J. Davern, W. Wesley Sutter, Hayley Mesch, Christopher Beinlich, Andrew Smith, Colton Ramstrom, Sarah White, Bryan Dubynsky, Kent Cantrell, Bill P. Hay, Weston Davis, Louis Mazzarelli

**Affiliations:** 1 Department of Veterinary Clinical Sciences, College of Veterinary Medicine, The Ohio State University, Columbus, OH, United States; 2 Tryon Equine Hospital, Charlotte, NC, United States; 3 Equine Athletic Veterinary Services, Simpsonville, KY, United States; 4 Woodland Run Equine Veterinary Facility, Grove City, OH, United States; 5 Peterson Smith Equine Hospital, Ocala, FL, United States; 6 Performance Veterinary Equine Services, Ocala, FL, United States; 7 Austin Equine Hospital, Driftwood, TX, United States; 8 Palm Beach Equine Clinic, Wellington, FL, United States; 9 Ocala Equine Hospital, Ocala, FL, United States; 10 UltraEquine Health, Glastonbury, CT, United States

**Keywords:** ligaments, equicenta CTM umbilical allograft, joints, prospective controlled clinical trial in lame horses, real world lameness clinical trial, tendonitis, standard of care, biologics

## Abstract

**Introduction:**

This multicenter, prospective, controlled clinical study and client survey evaluated the safety and effectiveness of an equine umbilical tissue suspension (equicenta® CTM; eCTM, 2:1) compared with clinician-selected standard of care (SOC) for treating lame performance horses.

**Methods:**

This pragmatic, real-world, open-label study enrolled horses diagnosed with appendicular musculoskeletal conditions affecting the joints, tendons, or ligaments under owner consent and an intention-to-treat design with an optional crossover after no improvement at 12 weeks and per-protocol *post hoc* analyses at 26 weeks. Horses were assigned to eCTM or clinician-selected, non-steroidal SOC; other case management was not different. Ordinal outcomes included lameness, pain, swelling, performance, adverse events, client-reported health, plus ultrasound and gait kinematics at baseline and at 2, 4, and 12 weeks, with veterinary remote follow-up at 26 weeks.

**Results:**

Enrolled horses (*n* = 138) completed 12 weeks, and 135 surviving horses completed 26 weeks. Cumulative improvement was greater for eCTM versus SOC by 2 weeks for pain and swelling, by 4 weeks for lameness, and through 12 weeks for all clinical outcomes (*p* < 0.001). Mean and cumulative performance scores were significantly greater for eCTM at 26 weeks, with the odds of returning to or exceeding prior performance favoring eCTM (2.72:1; *p* < 0.006). Client-reported body condition, hair coat, overall health, and quality-of-life scores were significantly better for eCTM (*p* < 0.03), and injection-related adverse events occurred more often in SOC, particularly for joint injections. Kinematic analysis showed significantly greater improvement in lameness with eCTM at impact and push-off.

**Discussion/conclusion:**

In this pragmatic setting, a single, off-the-shelf injection of eCTM was associated with earlier, greater, and more durable improvements in clinical and performance outcomes than clinician-selected non-steroidal SOC. The open-label design and flexible SOC comparators reflect real-world application of biologics and may have introduced expectation and treatment selection biases, although none were identified in post-sensitivity analyses. The magnitude and consistency of between-group differences across clinical, owner-reported, and kinematic outcome measures suggest an observed treatment effect. Overall, these positive findings suggest that eCTM may offer a single-dose alternative to clinician SOC approaches for selected musculoskeletal conditions in lame performance horses.

**Clinical trial registration:**

https://studypages.com/dashboard/ unique identifier is VCT #26005934.

## 1 Introduction

Clinical studies specifically examining lame performance horses are limited, even though lameness is a leading cause of poor performance, loss of use, and early retirement across disciplines (1–4). Musculoskeletal disorders of appendicular joints, tendons, and ligaments—including osteoarthritis, synovitis, suspensory desmitis, and tendonitis—are common in performance horse populations and frequently become chronic or recurrent (5–7). Despite advances in diagnostics and rehabilitation, many affected horses do not regain their previous level of work, and long-term management often entails substantial time, cost, and ongoing challenges for owners and veterinarians (1, 5, 8).

Over the last decade, intra-articular and intralesional biologic therapies, such as autologous hemoderivatives and other ortho-biologic products, have become widely used in equine sports medicine, particularly for musculoskeletal conditions of the joints and soft tissues, such as tendons and ligaments (9, 10). Despite technological advances, inter-animal variability in autologous products has made consistent composition and repeatable clinical responses elusive. Autologous products are inherently limited because they are derived from animals of variable health, often with comorbidities and impaired healing, which may constrain their beneficial effects (11–15). Equine performance practices routinely use injectable biologic therapies for these musculoskeletal conditions, yet there is limited peer-reviewed evidence regarding safety, donor consistency, product variability, or effectiveness for commercially available formulations. These limitations, combined with strong market interest in alternatives to autologous blood products, have stimulated the development of novel ortho-biologic strategies such as microparticulate umbilical tissue allografts for injection.

Cryopreserved umbilical-derived allografts represent a newer class of ortho-biologic treatment with the potential to modulate inflammation and support tissue remodeling through a combination of bioactive proteins and extracellular matrix components (16–20). Equicenta® CTM (eCTM) is a cryopreserved equine umbilical allograft designed for local injection into musculoskeletal tissues, specifically joints, tendons, and ligaments. Early studies on eCTM suggest potential clinical benefit, including improvement in clinical signs in a limited number of cases, biocompatibility *in vivo*, and biologic characterization *in vitro* (21, 22). Comparative prospective clinical data in performance horses are limited for any biologic, particularly under routine clinical practice conditions.

In contemporary equine sports medicine, orthobiologic therapies such as platelet-rich plasma (PRP), autologous conditioned serum (ACS), autologous protein solution (APS), and mesenchymal stromal cells are widely used to manage performance-limiting lameness, with injections commonly administered into both joints and soft-tissue structures (tendons and ligaments), often in horses that have multiple lesions within a limb or in more than one limb (5, 10, 23–26). Management decisions are influenced by practice patterns, client expectations, and prior treatment history, resulting in heterogeneous but clinically relevant case mixes. Comparative effectiveness studies of new ortho-biologic interventions that reflect this complexity may have advantages for application in the intended population and have some advantages over classical randomized clinical trials (RCTs) that focus more narrowly on a single lesion type or experimental models (27).

The primary objectives of this clinical study were to determine whether eCTM treatment was associated with greater improvement in clinical outcomes, defined as lameness, pain, and swelling, over 12 weeks, than SOC as used in current practice, and to compare safety profiles. Secondary objectives were to compare performance over 26 weeks, explore tissue-level remodeling and gait symmetry using quantitative ultrasound-based Tissue Remodeling Scores (TRS) and smartphone kinematic gait analysis.

Comparative analyses were conducted under an *a priori* superiority framework, in which the null hypothesis specified no difference between treatments and the alternative hypothesis specified that the test product would provide greater improvement than the comparator, in contrast to non-inferiority or equivalence frameworks. It was hypothesized that, in a real-world equine sports medicine setting, horses treated with eCTM would demonstrate greater and more durable improvement in clinical and performance outcomes than horses treated with clinician-selected SOC, with an acceptable safety profile, and support the hypothesis that structural remodeling metrics and kinematic gait analyses would align with these clinical findings.

## 2 Materials and methods

### 2.1 Study design and setting

This study is a real-world, pragmatic, prospective, multicenter, open-label, controlled clinical trial with a flexible comparator arm (28), conducted in lame performance horses under CONSORT guidelines (29), and was enrolled at eight equine specialty practices between 23 October 2024 and 31 August 2025. The trial compared a cryopreserved umbilical allograft (the intervention) with clinician-selected standard-of-care (SOC) treatments, with veterinary follow-up for 2, 4, and 12 weeks and remote veterinary follow-up at 26 weeks.

Analyses distinguished between horses, conditions, and injections. Horses were the unit of randomization, and conditions were the unit for clinical outcome analyses of lameness, pain, and swelling. Each enrolled condition received one primary injection at baseline. In the SOC arm, some biologic products had manufacturer recommendations for repeat injections; these protocol-defined repeat injections formed part of the primary management plan but did not generate new conditions and were not treated as independent observations for clinical outcomes. Rescue injections given after the 12-week assessment did not generate new outcome conditions but were included for a 26-week performance per-protocol analysis. For safety data, all injections, primary, planned repeats, and rescue injections, were counted in safety analyses so that the adverse-event profile reflected exposure at the injection level.

#### 2.1.1 Participating practices and veterinarians

Each practice had a designated site investigator (SI) veterinarian and one or two project veterinarians (PVs) as co-investigators. All were recognized specialty performance horse practices with experience in musculoskeletal diagnostics and biologic therapies. An independent external research consultant veterinarian (ALB) served as the multicenter study director, quality monitor, and third-party adjudicator.

SIs ensured that participating facilities met protocol requirements for space, technical support, record-keeping, imaging capabilities, and client management. Client, staff, or veterinarian complaints related to the trial were handled by the SI according to predefined procedures. Each practice aimed to enroll approximately 10–50 horses; all practices met the minimum target of 10 enrolled horses.

#### 2.1.2 Training and standardization

Before enrollment began, PVs underwent standardized training via interactive online sessions covering detailed protocol procedures, including randomization, kinematic video acquisition, ultrasound image collection, case report forms, logistics, and handling and storage of investigational product in mobile −80°C freezers (Sterling Ultracold Portable -80°C Freezer, Global Cooling Incorporated, 6000 Poston Road, Athens, OH 45701). At the study launch in each practice, the director attended in person for initial cases.

Consensus training in clinical scoring was performed using live cases to harmonize grading of lameness, pain on pressure and flexion, swelling, and adverse reactions, based on definitions in the protocol, with the goal of minimizing inter-observer variability across veterinarians and practices (30). Each veterinarian received hard-copy protocol binders and a password-protected tablet computer for electronic data entry. Once signed, electronic forms were locked and could only be modified by the responsible veterinarian.

Portable ultrasound units (Butterfly iQ3 Ultrasound System, Butterfly Operations Inc., 1,600 District Ave, Burlington, MA 01803, USA) were provided to each participating veterinarian, with images stored in a secure cloud environment and locked upon upload. Kinematic videos were captured on smartphones and analyzed within a dedicated clinical-trial account.

### 2.2 Eligibility and enrollment

Client-owned performance horses were eligible if they met all inclusion and exclusion criteria and had a current negative Coggins test. Owners or persons responsible signed informed consent and commitment forms before enrollment.

At enrollment, clients and veterinarians completed a detailed history form including prior treatments for the condition, discipline, peak level of performance, dates of onset, and performance limitations attributed to the condition.

Key inclusion criteria were appendicular musculoskeletal conditions of the diarthrodial joint or soft tissues (tendon or ligament) diagnosed as the source of pain and lameness and were anatomically assessable by ultrasonography; American Association of Equine Practitioners (AAEP) lameness grade 1–4 at the trot [0 = not lame at the walk and trot, 1 = not lame at the walk and intermittently lame at the trot, 2 = not lame at the walk and consistently lame at the trot under special circumstances such as lunging, 3 = not lame at the walk and consistently lame at the trot on a straight line, 4 = lame at the walk and trot on a straight line, and 5 = partially or fully non-weight bearing at the walk] at enrollment; body condition score 3–8; and no infectious disease, steroid administration, injection, or invasive procedures at the site of interest in the previous 30 days. Compliance with FEI and USEF medication rules for performance-affecting substances at baseline and at each evaluation was required. [Federation Equestra Internationale (FEI)/United States Equestrian Federation (USEF) Medications Rules and Regulations for International and National competition] [FEI Detection Times 2022, https://inside.fei.org].

Key exclusion criteria included recent systemic injection of any substance without director approval, local or systemic corticosteroid treatment at the site of interest within protocol-defined intervals, use of therapies targeting sensory nerves serving the site of interest, unhealed fractures in the joint within 2 months, non-appendicular conditions, breeding stallions, draft horses, ponies under 13.0 hands, and pregnant or lactating mares.

To be included, lameness had to be confirmed by diagnostic workup, such that the clinician was confident the condition(s) were the source of the lameness. Eligible horses were typically 2–20 years old; exceptions required director approval. Horses had to be otherwise healthy based on physical examination and general attitude.

More than one eligible condition per horse was permitted. Multiple conditions in the same limb (i.e., joint and periarticular ligament lesions), or bilateral conditions (i.e., bilateral coffin joint arthritis) could be enrolled, provided each contributed to lameness and met the inclusion criteria. In such cases, all treated conditions in each horse received the same assignment, either eCTM or SOC. Each condition was followed as a separate condition-level observation for outcomes. For conditions within the same limb, a single limb-level lameness score was assigned and applied to all relevant conditions, such that improvement required overall improvement of that limb, which represented a conservative criterion for response.

### 2.3 Interventions

#### 2.3.1 Umbilical allograft intervention arm

Equicenta® CTM was supplied as single-use vials containing 1.5 mL of a cryopreserved umbilical-derived protein and microparticle suspension. The product was shipped on dry ice and stored at −80 °C in dedicated freezers at each site until use. Immediately prior to injection, vials were thawed in the hand for 10–15 min, gently agitated to resuspend microparticles, and aspirated for aseptic administration. Joint injections were targeted as close as possible to the area of synovial thickening and suspected cartilage injury based on prior imaging. In ST horses, eCTM injection proceeded with needle placement relatively parallel to the fiber longitudinal orientation to deposit the dose along the epicenter of the fiber damage. eCTM was injected and distributed within the lesion as the needle was withdrawn.

#### 2.3.2 Standard of care comparator arm

For SOC, attending clinicians selected what they judged to be the best available treatment for that horse and specific musculoskeletal condition (SOC flexible arm). The goal was to reflect real-world use of hemoderivatives and other biologics and comparators in similar indications. All injections, including product type, dose, and site, were recorded. The nature of the clinician-selected standard of care typically would include ortho-biologics currently used to treat musculoskeletal conditions intra-articularly and intra-lesionally, such as autologous protein solution (APS), platelet-rich plasma (PRP), autologous conditioned serum (ACS), platelet-poor plasma (PPP), stem cells (expanded bone marrow- or fat-derived), amnion allograft, hyaluronic acid (HA), and polyacrylamide gels (PAAGs) (2.5% or 4%) (5, 10, 11, 15).

#### 2.3.3 Co-interventions allowed

At or before Day 0, each horse received an individualized management plan prescribed by the PV, reflecting routine practice for the specific condition. Plans could include ancillary medical procedures such as shock wave therapy and structured rehabilitation exercises. For SOC horses, the plan could include repeat biologic injections per manufacturer recommendations. Actual ancillary treatments and repeat injections were recorded at Day 0 and follow-up visits.

#### 2.3.4 Co-interventions not allowed

Steroid therapy, locally or systemically, was not allowed for the 12 weeks of primary outcome assessment for the study.

#### 2.3.5 Recording and categorizing

The same veterinarian who enrolled the case (with some exceptions) performed the Day 0 (baseline), 2-, 4-, and 12-week follow-ups. Clients, along with a study veterinarian, completed a 26-week survey assessment that included performance information. Owners or persons responsible documented veterinary therapies and rehabilitation on the follow-up forms.

Data were collected at predetermined time windows relative to the time of injection (Day 0); enrollment (−2 weeks to Day 0), 2 weeks (−3 days to +1 week), 4 weeks (−3 days to +2 weeks), 12 weeks (±4 weeks), and 26 weeks (±4 weeks). Out of 773 possible data collection points, 37 (4.8%) were outside the window, did not differ between eCTM and SOC, and were included in the intended time.

### 2.4 Allocation

Allocation was quasi-randomized within practices by initial coin toss and randomly allocated (Test(T)TC(Control), TCT, CTT) to achieve an ~ 2:1 eCTM versus SOC ratio. Allocation was not known at the time of case evaluation and was not predictable.

As with any random assignment, the ratio may not be achieved. Overall study allocation between groups was monitored centrally with the aim of achieving an overall allocation ratio of approximately 2:1 for eCTM to SOC. The final achieved ratio was 1.9:1 for both horses and conditions without any central intervention. Primary analyses included data from all practices.

### 2.5 Blinding and minimizing bias

This trial was conducted as an open-label study with blinding between practices but not within practices. Veterinarian, staff, and client blinding were upheld between practices for all data. Ultrasound quantitation and analyses were performed in a blinded manner. Data entry into spreadsheets from locked computers was performed by blinded personnel. Data analysts and statisticians were blinded to treatment for analyses, including kinematic data. Several measures were implemented to preserve internal validity and limit potential bias. Selection bias was limited by pre-defined eligibility, consecutive case enrollment, stratified blinding among practices, and the addition of quantitative secondary outcomes (31). Expectation bias was limited by standardized outcome definitions for lameness, pain, swelling, performance, and adverse events. PVs underwent consensus training in scoring lameness, pain on pressure and flexion, swelling, and adverse reactions according to protocol definitions, and the same PV typically performed all follow-up assessments for a given case.

Objective exploratory outcome measures, including quantitative ultrasound-based TRS and kinematic gait analyses, were obtained in subsets of horses to provide assessor-independent support for clinical findings.

Financial incentives were aligned and standardized to minimize performance and detection bias: participating veterinarians received no compensation or other benefits contingent on treatment selection or study outcomes. All veterinarians signed declarations confirming the absence of outcome-contingent financial relationships with manufacturers of products used in the study, including the sponsor’s product.

### 2.6 Outcome measures

#### 2.6.1 Primary outcomes

The primary outcome for effectiveness in this study was the change in AAEP-validated lameness grade (32, 33) and validated pain and swelling scores (34) at pre-specified time points of 2, 4, and 12 weeks. Each enrolled condition received one primary injection at Day 0, which formed the basis for all primary clinical outcome analyses of lameness, pain, and swelling at 2, 4, and 12 weeks.

The primary outcomes for safety were non-serious and serious adverse events requiring diagnostic workup or treatment, and acute worsening of lameness immediately after injection.

##### 2.6.1.1 Lameness evaluation

Lameness was graded using the AAEP lameness classification at enrollment and at weeks 0, 2, 4, and 12 by the PV. Lameness evaluations were performed and graded as the first step in a designated lameness area. Subsequently, horses were walked and trotted according to protocol, and trot sequences were recorded and uploaded to the kinematic analysis platform. Assignment of lameness grade was completed prior to evaluation of the gait video analyses. When indicated, horses were trotted after joint flexion for joint lesions or following palpation pressure over soft-tissue lesions. When indicated, horses were lunged to grade bilateral lameness.

##### 2.6.1.2 Pain and swelling scores

Pain to soft-tissue pressure at the lesion site or joint flexion was scored at each lameness evaluation on a 0–4 scale (21, 34, 35). Swelling of the joint or soft-tissue structure was evaluated visually and by ultrasonography at each lameness time point and scored 0–4 according to predefined criteria (34). Injection-site inflammation was scored separately on a 0–4 scale around the percutaneous injection site, based on predefined diameter and extent criteria, and contributed to the safety analysis of the adverse event profile (34). The approximate proximal–distal length of tendon or ligament lesions was also recorded.

##### 2.6.1.3 Safety outcomes

Safety outcomes were based on injection reactions (adverse events) reported by clients and confirmed and scored by veterinarians as non-serious or serious based on whether intervention, such as diagnostic tests or treatment, was applied. Veterinarians were requested to report and comment on their opinion as to whether the serious or non-serious event might be related to the product, as indicated by increased pain or swelling in or around the tissues or joint injected (21).

Inflammation at the percutaneous injection site was a separate outcome category, distinct from adverse events, and was collected along with clinical outcomes and per protocol to reflect a predefined score (0–4) for the size of skin and subcutaneous signs of inflammation at the needle injection site (21).

All Adverse Event reports were subsequently reviewed centrally by a blinded data analyst for any discrepancies between site classification and protocol definitions, and recommended reclassifications as per protocol. Reclassifications were reviewed and approved after a central independent veterinarian review. Sensitivity analyses explored the impact of reclassifying cases as possibly related to the product after a central independent veterinarian review. Both PV and central veterinary review outcomes were reported.

Additional injections at the condition site were permitted according to protocol. Two categories were defined: planned repeat SOC injections as part of the primary management plan for certain biologics, and rescue injections, either SOC or eCTM, allowed after the 12-week assessment for conditions that remained at least as lame as at baseline. All injections were assessed and included in safety data, including primary, prescribed repeat injections in the SOC controls, and rescue injections, including cross-over injections.

Planned repeat SOC injections and rescue injections did not create new outcome units but were recorded and included in safety analyses.

#### 2.6.2 Secondary outcomes

The secondary outcomes for this study were return-to-performance, subgroup analyses, rescue injection outcomes, owner/clinician assessments, imaging changes, and kinematic gait analysis.

##### 2.6.2.1 Performance assessment

At enrollment, discipline and peak competition level within the prior 6 months were recorded. At follow-up on at weeks 2, 4, 12 and 26 weeks, veterinarian performance scores from 1 to 4 were recorded in consultation with the owner or person responsible: 1 = no return to work or performance due to the injury; 2 = return to rehabilitation work without tack, but no return to performance; 3 = return to work under tack but not at prior performance level; 4 = return to or exceeding previous work or performance. Objective performance data were collected when available. Performance scores were assessed on an intention-to-treat basis.

##### 2.6.2.2 Client assessment

All owners or persons responsible with horses enrolled in the clinical study were simultaneously enrolled in a prospective client assessment study using a validated survey instrument administered at the same time windows as veterinarian follow-up at 2, 4, 12, and 26 weeks (34). Clients agreed to complete all forms prospectively at each scheduled time point, providing parallel data on comfort and health parameters.

##### 2.6.2.3 Rescue criteria and outcome assessment

Rescue injections were permitted only after the conclusion of the 12-week primary outcome assessment and included follow-up evaluations at 4 weeks and 12 weeks for lameness, pain, and swelling. Follow-up at 26 weeks of enrollment often coincided with the 12-week follow-up by the PV. The estimated study termination date of 31 March 2026 was 26 weeks after the last day of enrollment (31 August 2025) and included the option for a 26-week follow-up for many rescue horses (38 weeks) for comparable performance data (26 weeks) to primary injection.

For 26-week performance analyses, all rescued horses were retained in the intention-to-treat analysis according to their original randomized group and excluded from the per-protocol analysis. Both intent-to-treat and per-protocol results are reported. Rescue injections contributed to safety denominators and constituted new outcome units in prespecified per-protocol analyses only. *Post hoc* sensitivity analyses of parameters for this cohort of conditions for lameness, pain, and swelling were performed. Details on horses that failed to improve and were rescued with additional injections were tabulated and included limb, clinical diagnoses, original lameness score, and product.

##### 2.6.2.4 Computational ultrasound tissue remodeling score (TRS) and TRS validation

Ultrasound DICOM images of ligaments and tendons from day 0 to week 12 were downloaded from cloud or local systems, de-identified aside from horse, date, and condition ID, and blinded to treatment group. Images underwent quality review; 46 conditions met pre-specified image-quality criteria and were included in the exploratory tissue remodeling score (TRS) analysis. Regions of interest encompassing the lesion were manually selected, and six quantitative texture features were computed for each region of interest (ROI) using algorithms to derive a TRS, normalized to 50 at baseline for each horse and tracked over time. Each quantitative texture feature is based on quantification of recognized visual abnormalities in tendon or ligament (36), including structural measurements of central dark area and dark intensity, as well as quality measurements of fiber alignment and fiber echo intensity. Intra-rater segmentation reproducibility was assessed by cohort Dice analysis across 25 paired ROIs in eight cases spanning tendon and ligament conditions in the data set and compared to published intra-rater benchmarks for ultrasound segmentation (37). Validation of TRS to a clinical gold standard of lameness grade was assessed by within-horse correlation: ΔTRS to ΔLameness by Pearson Correlation at Day 14 and Day 30 in the tendon subset. TRS and performance outcome validity were assessed in ligaments and tendons to provide convergent evidence at the cohort level that the TRS treatment-effect signal aligns with an independent clinician-assigned performance measure (predictive validation). In conclusion, the segmentation step is reproducible enough that it does not account for the cohort-level treatment effect (ligament *d* = 0.97). TRS variability between segmentation passes is small relative to the eCTM-vs-SOC signal. All data will be considered secondary to primary outcomes and exploratory.

##### 2.6.2.5 Kinematic gait analysis

Kinematic gait analysis was performed using the dedicated motion-analysis platform (Sleip.com, Drottninggatan 92, 111 36 Stockholm, Sweden). For each time point, videos of trot and, when applicable, lunge were recorded, processed through a predefined data pipeline, filtered for quality, and summarized as asymmetry indices and related metrics. Recordings were assigned to the nearest follow-up window, with multiple recordings per time point averaged per horse.

### 2.7 Sample size and subgroup analyses

#### 2.7.1 Sample size

Sample size estimates for safety were based on limited allograft data reporting a 13% adverse reaction rate in 100 lame horses, so a minimum of 100 eCTM-treated horses was targeted to provide a reasonable safety profile (38). For the primary AAEP lameness outcome, a 1-grade improvement was considered highly clinically meaningful, with changes >0.5 grade also deemed important given the breadth of each ordinal grade. In prior randomized work in OA horses, lameness and client scores showed significant improvement with APS treatment compared with saline controls at *n* = 20 per group (35), and a separate allograft study in OA and soft tissue (desmitis/tendonitis) (*n* = 10 for OA and *n* = 10 for ST) demonstrated significant lameness improvement for soft tissue (ligaments/tendons) and near-significant improvement for OA (34). Based on the means and SDs from that work, with power 0.8 and alpha 0.05, a difference of 1 point in lameness and client scores required only three horses per group. Because our pragmatic clinical study was expected to show greater variability, we targeted approximately two-fold larger numbers of joints (*n* = 40/group or 80) and soft-tissue lesions (*n* = 20/group or 40) than in those studies, and it was anticipated to provide adequate power for the primary outcomes. For ultrasound and selected parameters, effect sizes (Cohen’s *d*) and Pearson’s *r* were used, with thresholds >0.75 or *r* ≈ 0.3 interpreted as large and clinically relevant. Odds ratios were calculated for key binary outcomes, such as becoming sound or pain-free (lameness and pain score of 0) and for adverse reactions, with alpha set at *p* < 0.05, while near-threshold values were sometimes reported when directionally consistent and clinically relevant. Given the heterogeneity of this pragmatic design, the goals were approximately 50 SOC and ≥100 eCTM conditions (2:1), and enrollment continued to the planned end date rather than stopping when these targets were reached.

#### 2.7.2 Subgroup analyses

As a preface, CONSORT and methodological papers emphasize that the primary analysis as designed (overall randomized comparison, allocation, and sample size assessments) should be maintained to protect the integrity of the primary, pre-specified analysis and remain the main basis for inference. This study’s primary findings are anchored to the prespecified inclusion of the overall population of musculoskeletal conditions, reflecting current practice. *Post hoc* subgroup analyses, while acknowledging the clinical interest, may be of lower credibility, mainly hypothesis-generating and recognized as exploratory in nature if the reduced sample size and increased variability underpower the comparisons. The primary objective of this pragmatic study was to compare equicenta® CTM versus clinician-selected standard care across musculoskeletal indications treated in current practice. The current application of the control comparator arm products across all tissues in equine practice provides a strong, practice-based rationale for focusing on the global effect on tissue types.

Explicitly, two predefined *post hoc* analyses were selected as clinically sensible contrasts for a limited, clinically driven exploratory subgroup analysis, tissue type, and SOC.

#### 2.7.3 Subgroup analysis by tissue type

The three musculoskeletal tissue types comprising the prespecified primary analysis included joints and soft tissues (tendons and ligaments). Each of these tissue types is often treated similarly with the same biologic and pharmaceutical products, administered by injection, such as biologics and steroids.

Joint, tendon, and ligament tissue subtype was compared between groups eCTM and SOC at 2, 4, 12, and 26 weeks for lameness, pain, swelling, and performance outcomes. Tendons were underpowered for subdivision due to a less robust subsample size; combining relevant outcomes of pain and lameness into a clinical index enhanced power. Findings are presented as exploratory, with emphasis on consistency of direction, not only statistical significance. These exploratory results, when analyzed this way, may be broadly consistent with the primary result.

##### 2.7.3.1 Subgroup analysis of the SOC by product class

The overall population study design reflects how hemoderivatives and other biologics are currently used, so the overall SOC class is the clinically relevant target population, rather than a collection of many separate, smaller studies. The current application of the comparator arm in equine practice provides a strong, practice-based rationale for focusing on the global effect of these ubiquitous blood proteins that have tissue-agnostic functions found in both eCTM and SOC.

Explicitly two predefined *post hoc* analyses were selected as clinically and statistically sensible contrasts for the SOC comparator arm; a subgroup analysis comparing overall hemoderivatives representing the largest subgroup in the SOC (*n* = 43; 63.2%) and the most popular biologics used in performance horse practice today, and the subgroup APS as the hemoderivative with the largest number of cases in this study (*n* = 22; 51% of the hemoderivatives) and with reported clinical efficacy data.

### 2.8 Statistical analysis

All statistical analyses were performed by an independent third-party biostatistician experienced in clinical trial analysis and reporting using R, primarily with the lme4, lmerTest, emmeans, and nlme packages. [R Core Team (2024). *R: A Language and Environment for Statistical Computing*. R Foundation for Statistical Computing, Vienna, Austria. URL https://www.R-project.org/] Clinical outcomes of interest included cumulative improvement from baseline in lameness, pain, and swelling scores at predefined time points, as well as 26-week performance scores.

#### 2.8.1 Analysis populations

The primary analyses of clinical outcomes up to 12 weeks were conducted according to the intention-to-treat principle, including all quasi-randomized horses in the treatment groups to which they were originally assigned. No horses withdrew or changed treatment assignment prior to the 12-week visit; therefore, intention-to-treat and per-protocol populations were identical for analyses through 12 weeks.

At 12 weeks, predefined rescue options permitted repeat injections or cross-over for horses that did not demonstrate improvement in lameness relative to baseline. For 26-week performance outcomes, two complementary analyses were performed: an intention-to-treat analysis including all quasi-randomized horses analyzed according to initial treatment assignment, reflecting real-world effectiveness, and a per-protocol analysis excluding horses that received rescue or cross-over treatment between weeks 12 and 26, providing an estimate of efficacy under the originally assigned regimen.

#### 2.8.2 Primary models for clinical outcomes

For the primary clinical outcome analyses, cumulative improvement in lameness, pain, and swelling scores, linear mixed-effects models were fitted to evaluate the effects of treatment group, time, and the treatment group × time interaction. To account for repeated measurements over time within the same treated condition, a random intercept for condition was included. Accounting for multiple conditions per horse did not materially alter the main clinical outcome findings. Outcomes were expressed as change from Day 0, which partially normalized baseline variability within each condition prior to modeling.

Estimated marginal means and 95% confidence intervals for each treatment group at each time point were obtained from the models, and planned pairwise contrasts between eCTM and SOC were calculated using the emmeans framework with Fisher’s least significant difference approach. Statistical significance was defined as *p* ≤ 0.05.

#### 2.8.3 Subgroup and sensitivity analyses

Prespecified analyses focused on the overall population of eligible musculoskeletal conditions treated with biologic injections, reflecting real-world use in participating practices. Subgroup and sensitivity analyses were considered *post hoc* and were exploratory and interpreted cautiously. Practice-related results were interpreted descriptively.

Secondary subgroup exploratory analyses were conducted within the same linear mixed-effects framework, stratified by condition category and, where numbers allowed, by tissue type (joint and ligament) separately. Additional exploratory analyses examined product-class subgroups within the SOC arm, for example, pooled autologous hemoderivative products and APS specifically, and compared those outcomes with the eCTM arm. Because these analyses were not prespecified and had smaller sample sizes, results were considered hypothesis-generating.

When appropriate, heterogeneous residual variance structures were specified to allow unequal variances across treatment groups and/or time points, particularly in subgroup models with reduced sample sizes. For frequency data, including lesion distributions, adverse-event frequencies, and the proportion of horses with more than one treated condition, Fisher’s exact tests were used. *p*-values for subgroup and sensitivity analyses were reported descriptively without formal adjustment for multiplicity, and interpretation emphasized consistency of direction and magnitude with the primary analysis rather than isolated nominal significance.

### 2.9 Ethical approval and consent

All procedures were approved by an independent third-party Institutional Animal Care and Use Committee before study initiation (ETCR-2024-0329; East Tennessee Clinical Research, Inc., Rockwood, TN, USA). Informed consent was obtained from 100% of owners or persons responsible for the horses before enrollment. The trial was registered in the AVMA Veterinary Clinical Trials Registry (VCT #26005934).

## 3 Results

### 3.1 Participant flow, recruitment, enrollment, and follow-up dates

**Figure 1:**
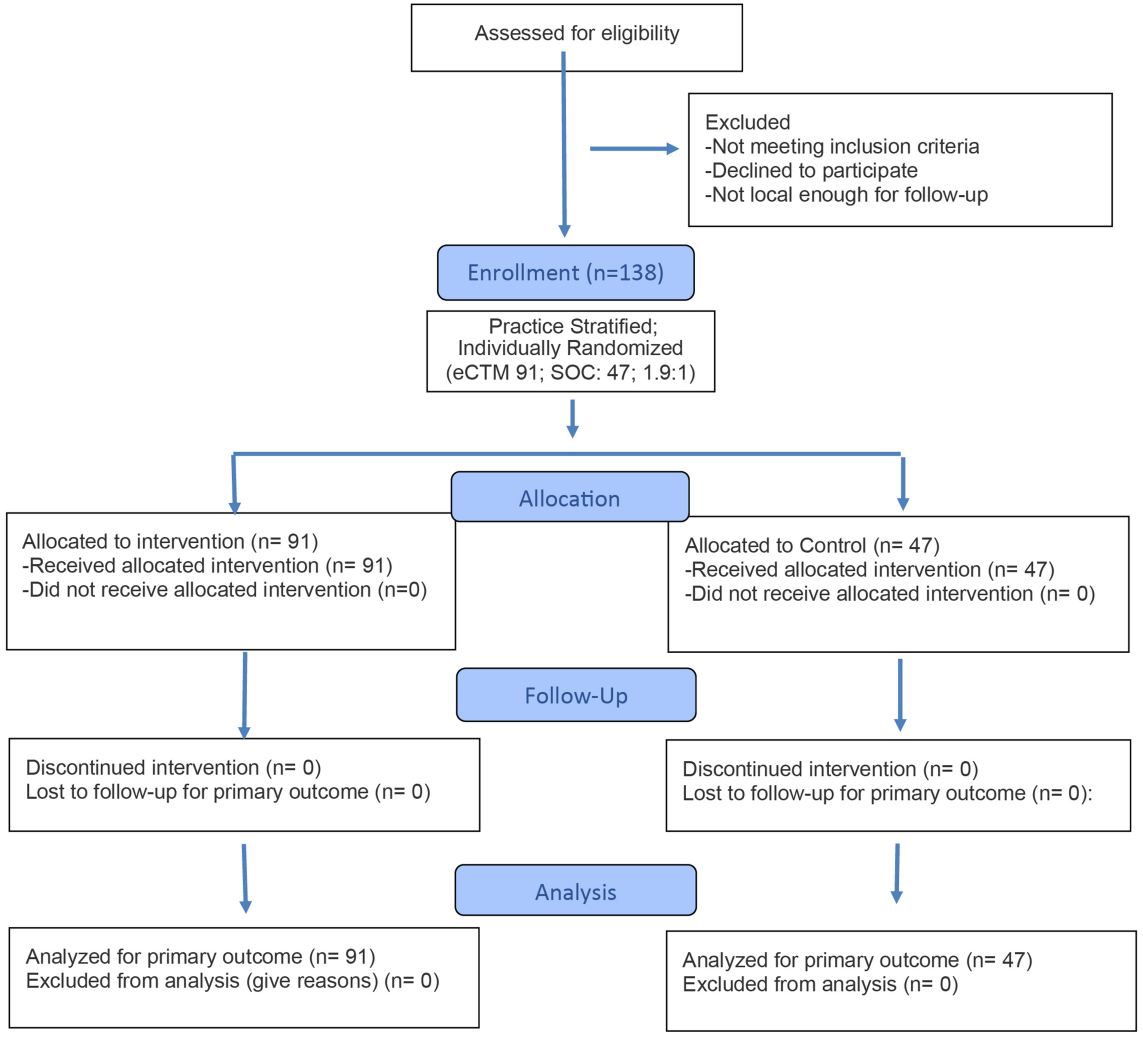
CONSORT-style diagram. The flow of horses and conditions through enrollment, allocation, follow-up, and analysis. Diagram modified from (64), licensed under CC BY 4.0.

All eight performance horse practices completed training, and the study, with 100% of enrolled horses, followed the 12-week veterinary assessment. A total of 138 client-owned performance horses were enrolled, contributing 195 eligible musculoskeletal conditions for outcome analyses. The flow of horses and conditions through enrollment, allocation, follow-up, and analysis is summarized in the CONSORT-style diagram (Figure 1).

Horses could be recruited at examination and returned for up to 2 weeks prior to starting the study on Day 0, the day of injection. Veterinary and client follow-ups occurred at 2, 4, and 12 weeks after Day 0.

All enrolled horses completed a 12-week veterinarian follow-up, and 135 of 138 horses (98%) completed the 26-week client and veterinarian performance assessment. Three horses died between weeks 12 and 26 due to colic, a racing accident, and acute neurologic disease; all were deemed unrelated to the study product by site veterinarians.

After enrollment ended on 31 August 2025 (study month 10). Follow-up by veterinarians was completed on 31 December 2025 for primary 12-week outcomes (Study month 14). Remote veterinary/client follow-up was completed on 31 March 2026 for 26-week performance outcomes and rescue injection 12-week outcomes.

### 3.2 Baseline characteristics and prespecified sensitivity analyses

All horses had a negative Coggins test, normal physical examinations, and baseline CBCs obtained in 99.3% of horses before enrollment. Follow-up CBCs were collected in 90.6% of horses, with missing samples distributed across groups with no significant difference between groups. All CBCs were considered acceptable by study veterinarians.

#### 3.2.1 Prior treatments and performance before enrollment

Most horses had experienced the target condition for less than 6 months at enrollment, and more than half in each group had received prior treatments that were insufficient to return the horse to full performance. The most common prior treatments were NSAIDs, biologics, and steroids, with similar distributions between groups, indicating comparable pre-study historical medical management (Table 1). Client-reported performance scores in the 6 months before enrollment did not differ between groups, indicating comparable pre-study performance levels.

**Table 1:** Prior veterinarian-prescribed treatments distributed by duration of condition recorded at enrollment and assigned to equicenta® CTM (eCTM) or standard of care (SOC).

Assigned group		Horses prior treatment (%)				
Pre-trial treatments		Total	<6 mo	6–12	12–24	>24
Pharma						
NSAIDs	*eCTM*	56.2	56.7	50.0	50.0	66.7
	*SOC*	55.6	47.6	75.0	0.0	100.0
Steroids	*eCTM*	27.1	6.7	50.0	50.0	83.3
	*SOC*	22.2	19.0	50.0	0.0	0.0
PSGAG	*eCTM*	12.5	10.0	10.0	0.0	33.3
	*SOC*	25.9	14.3	50.0	0.0	100.0
Biologic						
APS	*eCTM*	20.8	30.0	0.0	0.0	16.7
	*SOC*	33.3	38.1	25.0	0.0	0.0
PRP	*eCTM*	10.4	13.3	0.0	0.0	16.7
	*SOC*	25.9	23.8	25.0	0.0	50.0
HA	*eCTM*	8.3	6.7	10.0	50.0	0.0
	*SOC*	7.4	4.8	25.0	0.0	0.0
ACS	*eCTM*	0.0	0.0	0.0	0.0	0.0
	*SOC*	7.4	4.8	25.0	0.0	0.0
Amnion allograft	*eCTM*	12.5	13.3	20.0	0.0	0.0
	*SOC*	0.0	0.0	0.0	0.0	0.0
Stem cells	*eCTM*	2.1	3.3	0.0	0.0	0.0
	*SOC*	0.0	0.0	0.0	0.0	0.0
Platelet-poor plasma	*eCTM*	2.1	3.3	0.0	0.0	0.0
	*SOC*	7.4	4.8	25.0	0.0	0.0
Other bio	*eCTM*	6.2	6.7	10.0	0.0	0.0
	*SOC*	7.4	4.8	0.0	0.0	50.0
Physical therapy						
Shockwave	*eCTM*	6.2	6.7	10.0	0.0	0.0
	*SOC*	0.0	0.0	0.0	0.0	0.0
Surgery	*eCTM*	4.2	3.3	10.0	0.0	0.0
	*SOC*	0.0	0.0	0.0	0.0	0.0
Any prior Tx	*eCTM*	52.7	44.1	66.7	100.0	100.0
	*SOC*	57.4	55.3	66.7	0.0	66.7
No prior Tx	*eCTM*	47.3	55.9	33.3	0.0	0.0
	*SOC*	42.6	44.7	33.3	0.0	33.3
Duration	*eCTM*	100.0	74.7	16.5	2.2	6.6
	*SOC*	100.0	80.9	12.8	0.0	6.4
Total horses (n)	*eCTM*	91.0	68.0	15.0	2.0	6.0
	*SOC*	47.0	38.0	6.0	0.0	3.0

Distributions did not differ between eCTM and SOC.

#### 3.2.2 Diagnostic workup and enrollment diagnoses

The distribution of prior diagnostic work-up, including physical examinations, lameness examinations, diagnostic anesthesia (nerve and joint blocks), and imaging modalities, was similar with no significant differences between groups, indicating comparable pre-study diagnostic workup levels (Table 2). All horses underwent a complete diagnostic work-up, such that the PV was confident that the enrolled condition (diagnosis) was the primary cause of lameness. Distributions of specific diagnoses were generally similar between eCTM and SOC arms (Table 3).

**Table 2:** Veterinarian diagnostic tests for each horse were recorded at enrollment and assigned to equicenta® CTM (eCTM) or standard of care (SOC).

Diagnostic tests	eCTM (*n* = 91)		SOC (*n* = 47)	
	*n*	%	*n*	%
Physical examination	62	68.1	37	78.7
Lameness examination	76	83.5	42	89.4
Local anesthesia	28	30.8	18	38.3
Radiographs	43	47.3	29	61.7
Ultrasound	63	69.2	23	48.9
CT/MRI/NM	7	7.7	3	6.4
Other	1	1.1	0	0.0
Mean tests per horse	3.0		3.2	
Total tests recorded	280		152	

CT, computed tomography; MRI, magnetic resonance imaging; NM, nuclear medicine.

**Table 3:** Distribution of horses and musculoskeletal conditions by diagnosis and anatomic location assigned and treated by equicenta® CTM (eCTM) or standard of care (SOC).

Horses	eCTM (*n* = 91)		SOC (*n* = 47)	
Conditions	*n*	%	*n*	%
Arthritis/synovitis	44.0	34.4	32.0	47.8
Stifle	10.0	7.8	8.0	11.9
Fetlock	11.0	8.6	7.0	10.4
Coffin joint	6.0	4.7	7.0	10.4
Tarsus	3.0	2.3	2.0	3.0
Carpus	6.0	4.7	2.0	3.0
Hip	0.0	0.0	1.0	1.5
Unspecified joint	8.0	6.2	5.0	7.5
OCD	9.0	7.0	8.0	11.9
Stifle	7.0	5.5	7.0	10.4
Tarsus	2.0	1.6	0.0	0.0
Fetlock	0.0	0.0	1.0	1.5
Articular cysts	6.0	4.7	3.0	4.5
Stifle	4.0	3.1	2.0	3.0
Fetlock	1.0	0.8	0.0	0.0
Tarsus	0.0	0.0	1.0	1.5
Carpus	1.0	0.8	0.0	0.0
Tendonitis	22.0	17.2	6.0	9.0
SDF tendonitis	14.0	10.9	5.0	7.5
DDF tendonitis	8.0	6.2	1.0	1.5
Desmitis	47.0	36.7	18.0	26.9
Suspensory branch desmitis	22.0	17.2	6.0	9.0
Proximal suspensory desmitis	17.0	13.3	8.0	11.9
Collateral ligament desmitis	3.0	2.3	1.0	1.5
Desmitis	2.0	1.6	1.0	1.5
Annular ligament desmitis	1.0	0.8	1.0	1.5
Check ligament desmitis	2.0	1.6	0.0	0.0
Manica flexoria desmitis	0.0	0.0	1.0	1.5
Total conditions (195)	128.0	100.0	67.0	100.0

The distribution of diagnoses did not differ between eCTM and SOC.

OCD, osteochondrosis dissecans; SDF, superficial digital flexor tendon; DDF, deep digital flexor tendon.

#### 3.2.3 Performance discipline before enrollment

The distribution of prior performance discipline (dressage, jumping, racing, western, and other) did not differ between groups, indicating comparable types of performance expectations between groups (Table 4).

**Table 4:** Distribution of performance discipline by number of horses, musculoskeletal condition, and mean performance scores at 26 weeks for equicenta® CTM (eCTM) and standard of care (SOC).

Assigned group			Condition				Mean performance
Performance discipline		*n*	Ligament	Tendon	Joint	%	Week 26
Dressage	*SOC*	10.0	5.0	1.0	8.0	21.3	2.2
	*eCTM*	9.0	5.0	3.0	3.0	9.9	3.8
Eventing/hunter/jumper	*SOC*	13.0	6.0	0.0	14.0	27.7	2.5
	*eCTM*	16.0	5.0	5.0	10.0	17.6	3.4
Racing	*SOC*	14.0	1.0	3.0	17.0	29.8	3.3
	*eCTM*	36.0	23.0	4.0	26.0	39.6	3.6
Western	*SOC*	10.0	3.0	2.0	7.0	21.3	2.3
	*eCTM*	20.0	12.0	5.0	11.0	22.0	3.0
Other^*^	*SOC*	0.0	0.0	0.0	0.0	0.0	—
	*eCTM*	10.0	3.0	5.0	8.0	11.0	3.3
Grand total	*SOC*	47.0	15.0	6.0	46.0	100.0	2.7
	*eCTM*	91.0	48.0	22.0	58.0	100.0	3.4^**^ (*p* = 0.004)

^*^Competitive gaited, driving, and show horses other than hunter or jumper.

^**^Greater performance scores (*p* = 0.004) for equicenta® CTM (eCTM) compared to standard of care (SOC) at 26 weeks, with no significant difference by discipline.

#### 3.2.4 Demographic data at enrollment

Breeds represented were Thoroughbreds (39), Warmblood sport horses (33), Quarter Horses (33), Gaited Horses (6), Standardbreds (4), Arabians (4), and Morgan (1).

Age (mean 8.7 years), sex (89 males (including 28 stallions) and 49 females), baseline body weight (mean 490 kg), baseline body condition score (mean 5), affected limb (forelimb [49%] or hind limb [51%]) did not differ significantly between group assignment. Body weight and body scores are reported in Supplementary Tables S1 and S2.

#### 3.2.5 Practice-level recruitment and enrollment

Practices were selected near equestrian hubs with a density of performance horses to facilitate recruitment and diversity in the enrolled population and reflect variable breed, discipline, and potential musculoskeletal case diversity within a commutable distance for the project monitor. Enrollment volume of horses and conditions by practice locale and assignment to treatment group are summarized in Supplementary Table S3. As expected, practices differed in overall volume and case mix (range 12–53 conditions), such as Ohio, including racing Standardbreds, Kentucky, including racing and rehomed TBs, and Florida, including a mix of WB show horses, racing TBs, and western horses. As anticipated, some surgical practices that perform arthroscopy had a higher caseload in joints, and some sports medicine practices near rehabilitation training centers had a higher caseload in ligaments and tendons. Seasonal metrics contributed to the volume of recruitable cases, with Florida having a narrow window; however, all lesion categories were represented across the study and in every practice. Randomization occurred within practice, irrespective of condition, to manage musculoskeletal assignment writ large, rather than as a group of smaller independent studies that logistically would have been beyond the capabilities of this practice-based study.

### 3.3 Interventions received: horses, conditions, and injections

Horses were quasi-randomized to eCTM, 91 horses and 128 conditions, or SOC biologics, 47 horses and 67 conditions, yielding an achieved horse-level and condition-level allocation ratio of 1.9:1, close to the prespecified target (Table 5). The proportion of horses and distribution of more than one treated condition were identical in both groups, with no significant difference (*p* = 1.0), indicating comparable relative numbers of horses with more than one condition contributing to the outcomes (Supplementary Table S4). Specific treatments for SOC horses and co-interventions for SOC and eCTM horses are listed in Table 6.

**Table 5:** Distribution of the number of horses, musculoskeletal conditions, and injections for equicenta® CTM (eCTM) and standard of care (SOC) for the clinical study.

	equicenta® CTM	SOC control	Overall
Horses (*n*)	91	47	138
Conditions			
Joints	58	46	104
Ligaments	48	15	63
Tendons	22	6	28
Total conditions	128	67	195
Additional conditions, *n* (%)	37 (28.9%)	20 (29.9%)	57 (29.2%)
Additional injections			
SOC to SOC (7 prescribed+ 2 rescue)	0	9	9
SOC to eCTM (rescue-cross over)	0	11	11
eCTM to eCTM (rescue)	9	0	9
Total injections			
Joints	72	52	124
Ligaments	54	17	71
Tendons	22	7	29
Total	148	76	224
Additional injections, *n* (%)	20 (13.5%)	9 (11.8%)	29 (12.9%)

SOC horses had significantly more additional injections than eCTM horses (*p* < 0.03).

There was no difference between SOC and eCTM in the number of injections for rescue horses (*p* > 0.09).

**Table 6:** Distribution of assigned treatment administered by horse for equicenta® CTM or standard of care (SOC) to horses in the clinical trial.

Trial treatment	eCTM		SOC	
	*n*	%	*n*	%
Synthetic non-biologic	0.0	0.0	5.0	10.6
Hyaluronic acid	0.0	0.0	3.0	6.4
Polyacrylamide hydrogel	0.0	0.0	2.0	4.3
Biologic	91.0	100.0	42.0	89.4
Amnion allograft	0.0	0.0	4.0	8.5
Autologous conditioned serum (ACS)	0.0	0.0	4.0	8.5
Autologous protein solution (APS)	0.0	0.0	15.0	31.9
Bone marrow aspirate	0.0	0.0	1.0	2.1
equicenta® CTM	91.0	100.0	0.0	0.0
Exosomes	0.0	0.0	2.0	4.3
Platelet-rich plasma (PRP)	0.0	0.0	11.0	23.4
Platelet-poor plasma (PPP)	0.0	0.0	3.0	6.4
Stem cells - adipose	0.0	0.0	1.0	2.1
Stem cells (bone marrow)	0.0	0.0	4.0	8.5
Physical therapy (ancillary)	43.0	47.3	19.0	40.4
Shockwave	18.0	19.8	11.0	23.4
Rehabilitation	25.0	27.5	8.0	17.0
Total horses	91.0	100.0	47.0	100.0
Total	134.0	147.3	69.0	146.8

Physical therapy did not differ between SOC control and equicenta® CTM.

Across the study, 224 injections were administered, consisting of 195 primary injections, one per enrolled condition, plus planned repeat SOC injections (7 injections) and rescue injections (22 injections) (Table 5). A significantly greater number of re-injections occurred in SOC (*p* < 0.03), in part due to the repeat prescribed injections (7 injections) for some products. The number of rescue injections did not significantly differ between SOC and eCTM horses (*p* > 0.09). Clinical outcome analyses of lameness, pain, and swelling were based on the 195 enrolled conditions, whereas safety analyses were based on all 224 injections.

### 3.4 Outcome measures

#### 3.4.1 Primary outcomes

##### 3.4.1.1 Clinical outcomes

**Figure 2:**
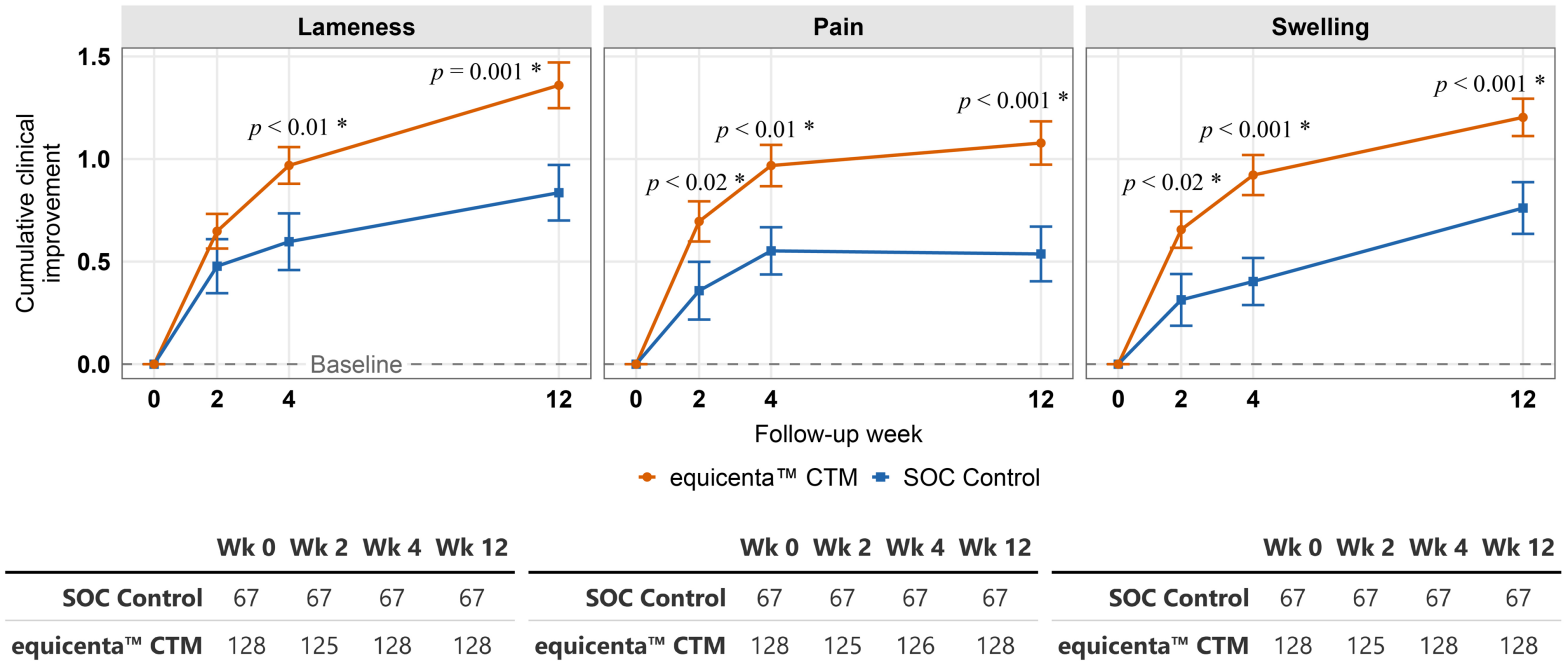
Cumulative clinical improvement trajectories from baseline score (mean ± SEM) for the clinical outcomes of lameness, pain, and swelling recorded by study veterinarians for 195 conditions over 12 weeks for equicenta® CTM or standard of care (SOC) control. Follow-up was 98–100% for all weeks as reported in the tables below the figure. Cumulative improvement was statistically greater for equicenta® CTM compared to standard of care (SOC) by 2 weeks for pain (*p* < 0.05) and swelling (*p* < 0.03), 4 weeks for lameness (*p* < 0.02) and out to 12 weeks (*p* < 0.003) for all clinical outcomes.

As defined by the study design, primary clinical outcomes are lameness, pain, and swelling of musculoskeletal conditions, quasi-randomized contemporaneously within practice to follow the usual standard of care of current biologics applications. In linear mixed-effects models of cumulative improvement from baseline, clinical conditions showed significant improvement in lameness, pain, and swelling scores over time (*p* < 0.001). Conditions treated with eCTM had significantly greater cumulative improvement than SOC, with between-group differences emerging by week 4 for lameness (*p* < 0.01) and by week 2 for pain and swelling (*p* < 0.02) and persisting through week 12 for all clinical outcomes (*p* < 0.001; Figure 2). Frequency analyses demonstrated that by week 12, the odds for eCTM to achieve a lameness score of 0 (sound) at >1.6:1 (*p* < 0.06) and a pain score of 0 (no pain) at 3:1 (*p* < 0.001) were greater than SOC.

##### 3.4.1.2 Safety outcomes and adverse reactions

Percutaneous inflammation at the injection sites was scored 0 (no inflammation) by veterinarians at all-time points for all conditions in both eCTM and SOC.

Primary safety outcomes included adverse reactions and increased lameness occurring within a time frame potentially associated with the product (1–2 days). Central review identified more cases meeting the criteria for serious and possibly related to the product more often than veterinarians. Regardless, in both veterinary and central review, 4.8:1 (*p* < 0.04) and 5.9:1 (*p* < 0 = 0.002), respectively, a significantly greater number of injection reactions were reported in SOC than eCTM. On an injection basis and persistence of the reaction, this corresponded to a greater burden of adverse-event reports for SOC than eCTM. In the SOC arm, PAAG was overrepresented in adverse events and occurred in joints. When PAAG was removed from the data set, SOC still had greater odds of a reported adverse reaction than eCTM (3.4:1; *p* < 0.03). Details of adverse reaction, including practice, product, breed, discipline, diagnosis, clinical signs, final lameness score, and performance outcome, are reported in Supplementary Table S5. No obvious trends for common demographics or effects on long-term outcomes were noted. No adverse event was considered to represent infection by the site veterinarian or central review. Overall reported reactions were 3.4% of eCTM injections (5 of 148) and 10.5%% of SOC injections (8 of 76) without PAAG reactions included.

#### 3.4.2 Secondary outcomes

##### 3.4.2.1 Performance outcomes

**Figure 3:**
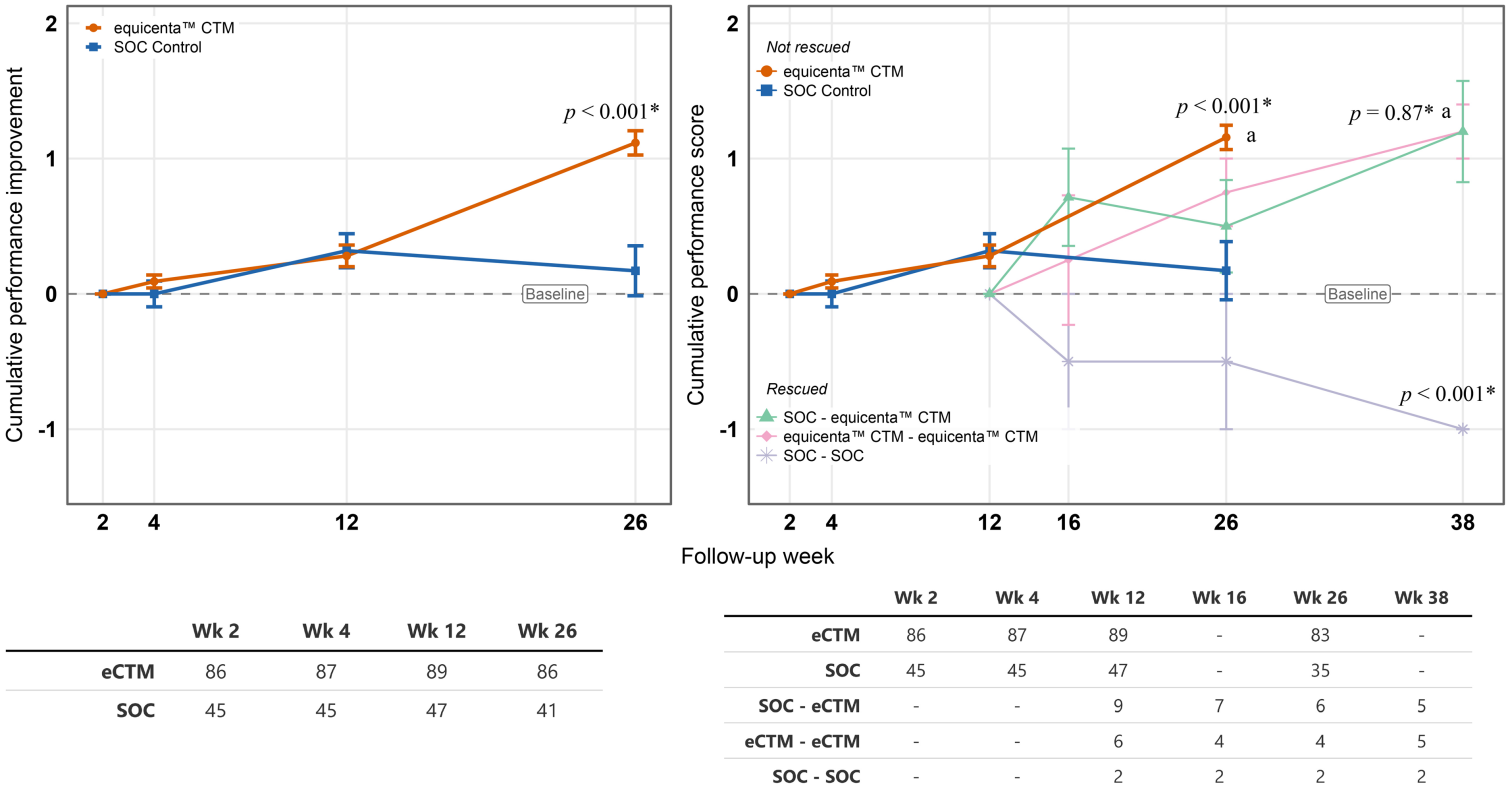
Cumulative performance trajectories (mean ± SEM) for horses from baseline score recorded over 26 weeks by veterinarians and/or clients for equicenta® CTM or standard of care (SOC) control. Follow-up was >95% for 12 weeks as reported in the tables below the figure. Cumulative performance improvement was statistically greater for equicenta® CTM compared to standard of care (SOC) by 26 weeks (*p* < 0.001) for both intent-to-treat (left) and per protocol analyses (right). After 12 weeks, 19 horses (13%) with performance follow-up did not improve from week 0 and opted to receive rescue injections as per protocol, resulting in three rescue groups followed for an additional 4, 12, and 26 weeks. SOC horses that received a rescue injection of a different SOC steadily declined in cumulative performance over the next 26 weeks and differed from other rescue horses (*p* < 0.001). The SOC and equicenta® CTM horses that received rescue equicenta® CTM demonstrated cumulative performance improvement over the next 4, 12, and 26 weeks, such that equicenta® CTM rescued horses did not differ (a to a; *p* = 0.87) compared to the not-rescued equicenta® CTM population by 26 weeks after rescue injection.

Performance analyses on all horses assigned to groups at the start of the study, without correction for loss of horses during the study or crossover cases, reflect the intent-to-treat design for real-world effectiveness. The magnitude and direction of treatment effects on 26-week performance were consistent with the intention-to-treat analyses, with eCTM maintaining a significant advantage over SOC (Cohen’s *d* 2.23; *p* < 0.001; Figure 3). On average, SOC horses had returned to work or rehabilitation but not to prior performance levels, whereas eCTM horses, on average, had returned to work under tack and rider or sulky at or near their prior level (Table 4).

A greater proportion of eCTM horses achieved a performance score of 4, defined as return to or exceeding previous performance (79%), than SOC (21%) horses. The odds of returning to or exceeding prior performance were higher with eCTM than SOC (2.72:1: *p* < 0.006). In the per-protocol analysis that contained rescue horses, the magnitude and direction were consistent with the intent-to-treat analysis, with eCTM horses having significantly greater improvement in performance than SOC horses (*p* < 0.001; Figure 3). SOC horses that remained on SOC after rescue showed a decline in performance scores over time. In contrast, SOC horses that crossed over to eCTM and eCTM horses that received repeat eCTM had 26-week performance scores that were not significantly different from those of horses treated with a single primary eCTM injection, whether rescue horses were included or excluded from the analysis. Thus, horses rescued with eCTM appeared to respond similarly to horses that received eCTM as their initial treatment.

In *post hoc* subgroup analyses, the cumulative improvement in performance is most pronounced and sustained in eCTM ligaments, whereas SOC ligaments failed to thrive in performance by 26 weeks (See section 3.4.2.6).

##### 3.4.2.2 Client-reported outcomes

**Figure 4:**
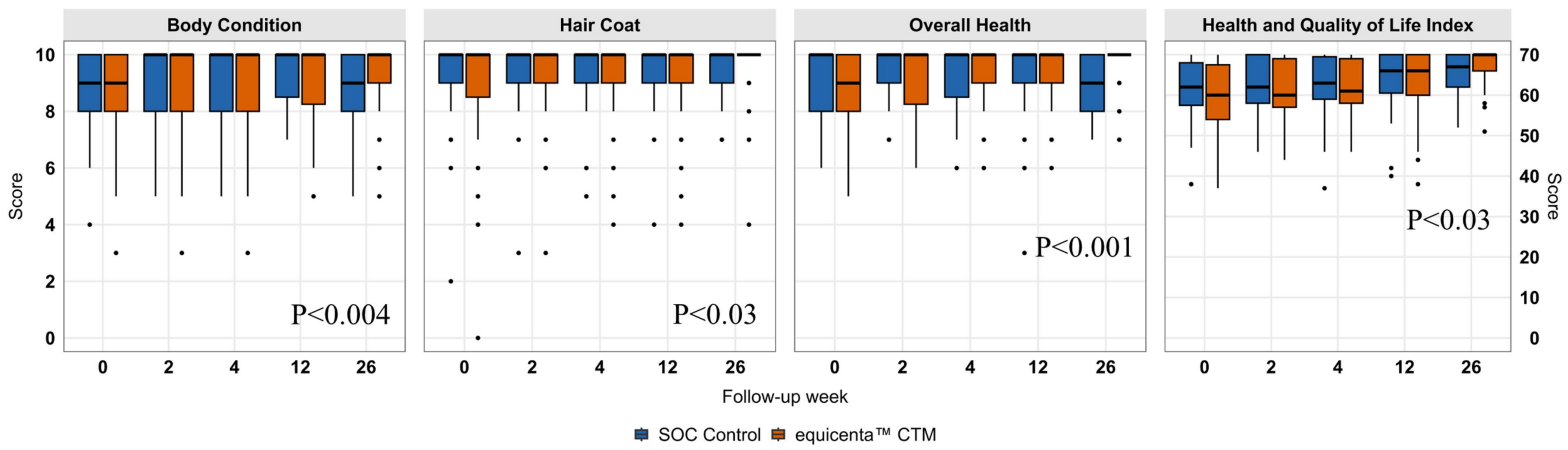
Client assessment scores (0–10) at baseline, 2, 4, 12, and 26 weeks of the clinical study for seven categories of health and comfort. Box-and-whisker plots showed that scores significantly increased in all categories and became less variable across time, reflecting improvement in health and comfort over the 26 weeks (*p* < 0.001). For the categories of body condition (*p* < 0.004), hair coat (*p* < 0.029), overall health (*p* < 0.001), and an index (sum of all seven categories) (*p* < 0.03), scores were significantly greater for equicenta® CTM (eCTM) than SOC control at 26 weeks reflecting an elevated client perception of their horses health and quality of life after eCTM compared to SOC control. Comfort in the stall, comfort at turnout, appetite, and attitude did not differ between eCTM and SOC control.

Client participation was high, with full completion of assessments at all scheduled time points except for the three deceased horses at 26 weeks. Owner-reported scores and the composite Health and Quality of Life Index improved significantly over time in the overall cohort, with decreasing variability across time (Figure 4). At 26 weeks, scores for body condition, hair coat, overall health, and the total Quality of Life Index were higher in eCTM horses than in SOC horses, indicating greater perceived health and quality of life after eCTM treatment. Client-reported comfort in the stall, comfort at turnout, appetite, and attitude improved over time but did not differ significantly between groups.

##### 3.4.2.3 Additional injections and rescue treatment

Total injections included prescribed additional injections of specific SOC and rescue injections in addition to the primary injections (Table 5).

Across all practices, 19 horses met the prespecified rescue criterion of no improvement in lameness at 12 weeks and received rescue injections between weeks 12 and 26 (Table 5). Rescue injections were administered only after completion of the 12-week assessments and therefore did not affect 12-week lameness, pain, or swelling analyses. In the SOC arm, two horses received a different SOC treatment at the same site, and 10 horses crossed over to eCTM (11 injections); in the eCTM arm, seven horses received 9 repeat eCTM injections. There was no significant difference between groups in the proportion of additional injections (*p* > 0.09), but there was a significant difference in the proportion of total additional injections in SOC horses compared to eCTM horses due to the addition of prescribed repeat injections (*p* < 0.03). Detailed characteristics of horses receiving rescue injections are provided in Supplementary Table S6.

##### 3.4.2.4 Ultrasound tissue remodeling score

**Figure 5:**
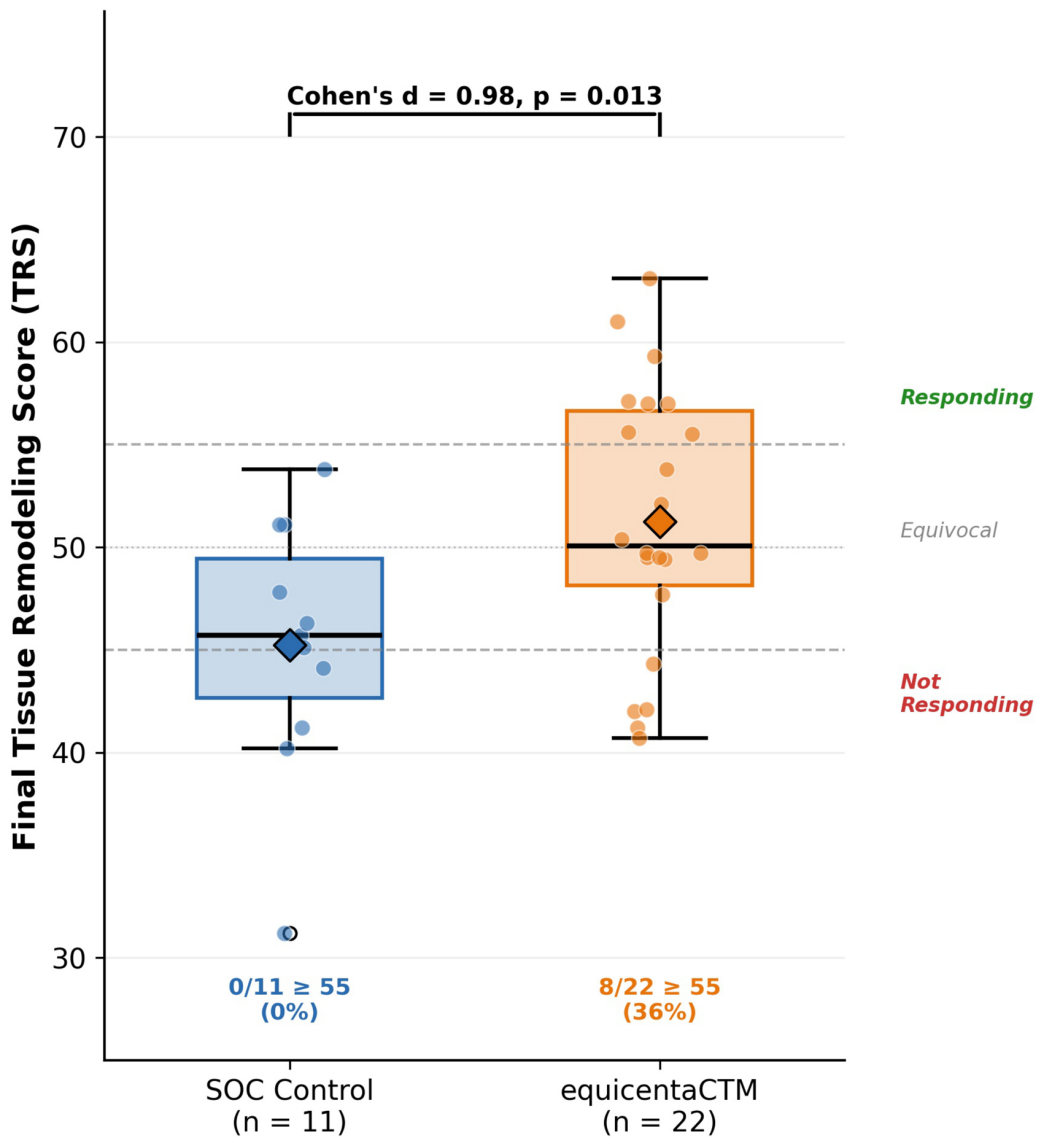
Box-and-whisker plots of final Tissue Remodeling Score (TRS) for ligament in standard-of-care (SOC) control (*n* = 11, blue) and equicenta® CTM (eCTM) (*n* = 22, orange) groups. Horizontal dashed lines indicate TRS classification thresholds: ≥ 55 = Responding, 45–55 = Equivocal, < 45 = Not Responding. Diamonds represent group means; individual data points are shown with jitter. No SOC control ligament (0/11, 0%) reached the Responding threshold (≥ 55) at final assessment, compared with 8 of 22 (36%) eCTM ligaments (Cohen’s *d* = 0.98, *p* = 0.013).

A subset of horses and conditions had ultrasound images of sufficient quality at baseline and follow-up to undergo quantitative TRS analysis. Ligament conditions treated with eCTM showed significant improvement in TRS from baseline, whereas ligament conditions in SOC horses declined, suggesting the hypothesis that there is a between-group effect size (Figure 5). A responding computational ultrasound Tissue Remodeling Score occurred in 36% of eCTM versus 0% of SOC ligaments by 12 weeks (*p* < 0.013). Proximal suspensory ligament lesions showed the largest treatment effect (*d* = 1.25), followed by lateral suspensory branch lesions (*d* = 0.82). The tendon sub-cohort (*n* = 13) was underpowered for group comparison (*d* = 0.14). Detailed ligament TRS trajectories are presented as graphics, including 3-D images of proximal suspensory lesions over time for a typical eCTM responder ligament and a SOC deteriorating ligament for tissue response analysis (Supplementary Figure S1).

TRS is a novel quantitative ultrasound metric and was used here as an exploratory structural endpoint. Inter-pass segmentation consistency was high, with a cohort mean Dice coefficient across 25 paired regions of interest from eight cases of 0.931 (range 0.843–0.947). Tendon TRS correlated significantly with change in limb-level lameness at 2 and 4 weeks (*r* − 0.74; *p* < 0.02), supporting the sensitivity of within-horse TRS changes to clinical status. The TRS trajectory was convergent with the hypothesis of an eCTM treatment effect, strongest for ligaments (*d* = 0.96; *p* < 0.073), and was associated with 26-week performance outcomes (Supplementary Figure S2).

Improvement in two-week TRS correlated strongly with final TRS (*r* = 0.88).

##### 3.4.2.5 Kinematic gait analysis

**Figure 6:**
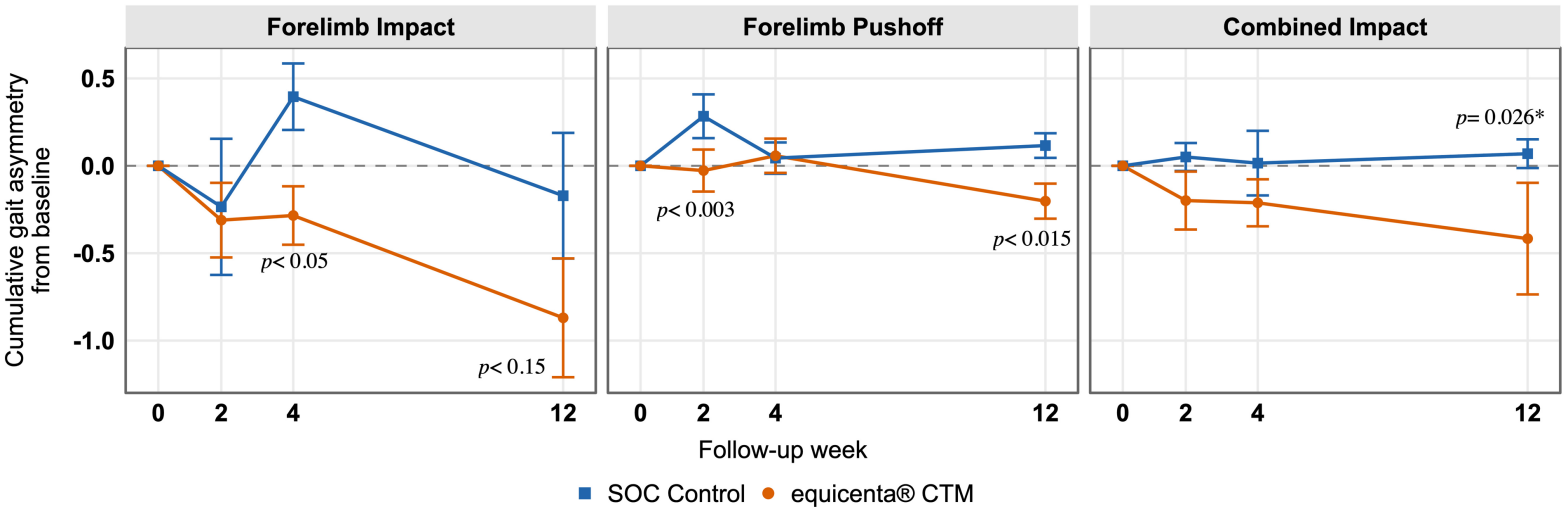
Cumulative improvement in forelimb lameness for eCTM compared to SOC for impact and push-off from individual video asymmetry analyses (Sleip.com). Horses receiving eCTM showed steady improvement (lower values) in lameness over 12 weeks that were statistically significant for impact at 4 weeks (*p* < 0.05) and push-off at 2 (*p* < 0.003) and 12 (*p* < 0.01) weeks compared to SOC. HIndlimb combined with forelimb cumulative improvement in lameness was more variable than forelimb but eCTM was still favored over SOC at 12 weeks (*p* < 0/026).

Kinematic data were available for a subset of horses after quality filtering. The most consistent and least variable data were obtained for forelimb lameness, where eCTM demonstrated greater cumulative improvement in asymmetry indices than SOC, particularly for impact at 4 weeks and push-off at 2 and 12 weeks. Combining hindlimb and forelimb data was more variable but still favored eCTM at 12 weeks for impact (*p* < 0.026) (Figure 6). Hindlimb and combined-limb data were highly variable and underpowered for further between-group analysis.

##### 3.4.2.6 Subgroup outcomes

**Figure 7:**
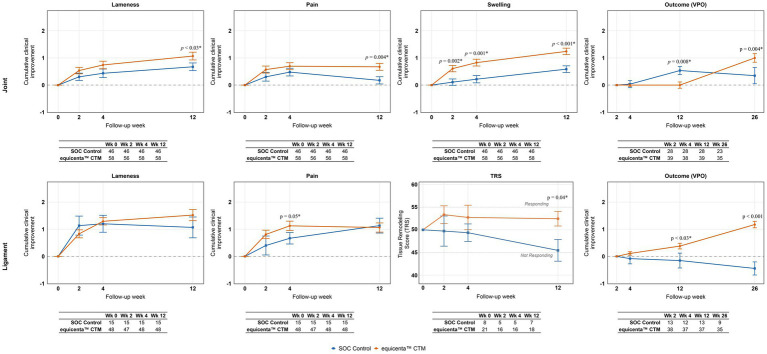
Subgroup analysis for conditions: cumulative clinical improvement trajectories from baseline score (mean ± SEM) for the outcomes of clinical lameness, pain, swelling, and veterinary performance outcomes (VPO) over 12 weeks for equicenta® CTM or standard of care (SOC) control for joints (top row) and ligaments (bottom row). For joints, eCTM had significantly greater improvement in lameness, pain, and swelling at multiple time points and at 12 weeks (*p* range <0.03 to <0.001). SOC had better improvement in performance at 12 weeks (*p* < 0.008), which subsequently deteriorated, as did the original improvement in joint pain. By 12 weeks, eCTM had greater improvement in performance for joints than SOC (*p* < 0.004), showing the durability of the umbilical tissue allograft treatment. The soluble proteins in SOC blood products are expected to be shorter-lived but also showed lesser improvement in lameness and pain than eCTM. For ligaments (bottom row), numerically the cumulative improvement scores were greater for eCTM than SOC at most time points and significantly for pain at 4 weeks. In both groups, the lameness trajectory for ligaments was flat after 2 weeks, indicating a slow clinical lameness response with ligament conditions. Ultrasound tissue remodeling score TRS showed significant divergence favoring eCTM by 12 weeks (*p* < 0.04), translating to greater performance at 26 weeks than SOC (*p* < 0.001). In concert, eCTM improved some clinical parameters earlier in healing, had better ultrasonographic remodeling, and ultimately greater performance within 26 weeks than SOC, showing a lag in effect compared to joints and tendons and requiring 26 weeks to translate to return of performance capabilities. Tendon was underpowered for subgroup analyses; however, directional changes favored eCTM but did not differ (*p* < 0.08). Data not shown.

Condition-specific trajectories for joints and ligaments based on cumulative change from baseline, with each horse serving as its own control, are shown in Figure 7.

The largest directional group differences are seen for joints; eCTM reflected greater cumulative improvement in lameness, pain, and swelling than SOC by 12 weeks and a more durable effect on performance at 26 weeks (range of *p* < 0.03 to <0.001; Figure 7). Joint-specific subgroup analyses revealed that SOC-treated joints showed better improvement in performance at 12 weeks than eCTM joints without corresponding sustained improvement in joint pain, followed by a decline in performance between 12 and 26 weeks. In contrast, eCTM joints exhibited significant improvement in performance by 26 weeks, preceded by improved joint pain at 12 weeks. Joints have a sufficient number of conditions per group for analyses.

The ligament-specific subgroup analysis must be interpreted with caution due to the smaller subgroup size and corresponding increase in variability; however, similar directional patterns in favor of eCTM persist, and eCTM remained numerically greater at most time points to SOC in lameness and pain, with a statistically significant improvement in pain at 4 weeks. The ligament TRS trajectory revealed that TRS in the ligament was significantly greater (responding) in the eCTM group, suggesting the hypothesis that eCTM had more ligament tissue remodeling than the SOC at 12 weeks. The tendon-specific subgroup analysis was underpowered due to a lack of robust numbers.

##### 3.4.2.7 Product-class subgroup analyses

**Figure 8:**
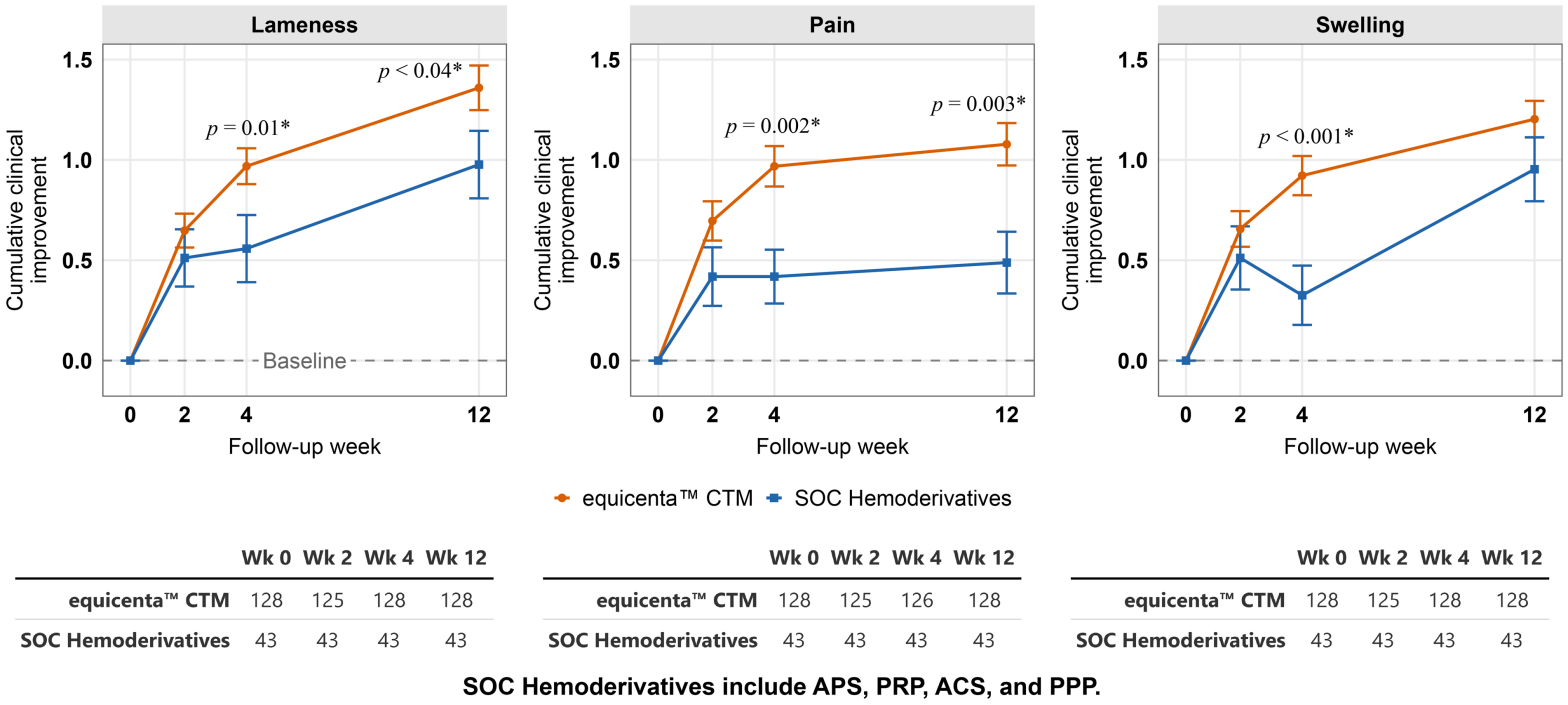
Sub-class analysis for hemoderivatives. Cumulative clinical improvement trajectories from baseline score (mean ± SEM) were greater for eCTM than the SOC subclass of hemoderivatives for the outcomes of clinical lameness, pain, and swelling at most timepoints over 12 weeks, particularly for lameness (weeks 4 and 12; *p* < 0.04) and pain (*p* < 0.003). Hemoderivatives consisted of APS, PRP, ACS, and PPP.

Because standard-of-care biologics in this trial contained several products in the same class and comprised a majority of the SOC (64.2%), a *post hoc* sub-product-class analysis was performed. Cumulative lameness, pain, and swelling trajectories for eCTM versus pooled data from autologous hemoderivative products, including APS, PRP, ACS, or PPP, are shown in Figure 8. Across multiple time points, eCTM demonstrated greater improvement (*p* < 0.04 to *p* < 0.001) than pooled hemoderivatives, suggesting the hypothesis that eCTM may be associated with improved lameness, pain, and swelling compared to the hemoderivatives as a subclass.

In an exploratory subgroup analysis, APS was selected to compare to eCTM because APS was the hemoderivative with the largest use (*n* = 22; 51% of hemoderivatives) in this product class and had prior supportive evidence in equine joint osteoarthritis (35). eCTM remained associated with multiple time points of greater improvement, primarily in lameness and pain, although this analysis was not prespecified and was limited by the smaller sample size, which limited the conclusions (Supplementary Figure S3).

## 4 Discussion

This pragmatic, multicenter, controlled clinical trial in real-world performance horses with musculoskeletal lameness is the first to show that treatment with locally administered umbilical proteins and micronized tissue particles (equicenta® CTM) was associated with greater and more durable improvement in clinical and performance outcomes than clinician-selected SOC biologic therapies. Across the musculoskeletal conditions of the joints and soft tissues, including tendons and ligaments, eCTM-treated conditions showed earlier divergence and larger cumulative improvements in lameness, pain, and swelling scores than SOC, with differences emerging by 2–4 weeks and persisting for at least 12 weeks. At 26 weeks, horses assigned to eCTM had higher veterinarian and client performance scores and greater odds of returning to or exceeding prior performance levels than SOC horses, in both intention-to-treat and per-protocol analyses. These clinical findings were supported by quantitative kinematic and exploratory ultrasound data. Collectively, these positive results, together with a satisfactory safety profile, support the use of equicenta® CTM as an alternative therapy for selected musculoskeletal conditions in performance horses.

### 4.1 Clinical effectiveness and durability of response

Conditions treated with eCTM were associated with greater cumulative clinical improvement. This association, based on timing, reflects an earlier clinical recovery and extended duration of benefit, consistent with known properties of the allograft (21). Potential benefits from hemoderivatives would be anticipated to be relatively short-lived because soluble proteins are rapidly absorbed or degraded (40), and one controlled clinical study showed APS benefit for osteoarthritis out to 2 weeks, with longer-term outcomes unknown (35). Umbilical allograft (eCTM), however, delivers fetal anabolic and other reparative proteins (21, 22), in part as a biodegradable connective tissue, releasing proteins over time, and may contribute to a plausible mechanism for the sustained benefit observed. The uniformity of the positive response across musculoskeletal tissues (joints, tendons, and ligaments) was anticipated. Many biological processes are shared among musculoskeletal connective tissues and are the established rationale for using biologics in these same tissues (41–45). Early use of biologics, before macroscopic structural pathology, remains an aspirational goal, targeting early interstitial changes such as glycosaminoglycan loss in cartilage (46). While biologics may not architecturally repair established physical defects such as cartilage, biologics may support interstitial tissue health and repair, promoting endogenous cellular ingrowth and hydroscopic properties (46, 47), analogous in scope—though not in mechanism—to the broad, cross-tissue effects observed with corticosteroids (48, 49). Overall, the therapeutic objective is to enhance connective tissue resilience and repair by delivering treatment when and where it will have maximal impact.

Performance trajectories reinforced the clinical findings. Similar rehabilitation recommendations (Figure 7; Table 6), especially for slower-healing tendon and ligament lesions, likely contributed to the initial lag in performance divergence in both groups. Between 12 and 26 weeks, SOC performance gains plateaued or declined, particularly in soft-tissue conditions, and, although exploratory, aligned with the poorer ultrasound tissue quality scores, whereas eCTM horses continued to improve in performance. These data suggest that eCTM may help sustain functional recovery.

Clinical and performance recovery are recognized as distinct domains (50); return to sport or work depends on structural healing and resolution of pain so that progressive conditioning and discipline-specific retraining can begin. The 26-week endpoint integrated recovery in a short enough timeframe to reflect the impact of treatment, permitting earlier sport training in eCTM horses (Table 4). A prior study of stifle arthroscopy cases with chondromalacia treated with bone marrow-derived mesenchymal stem cells, equine amniotic allograft, or no product did not demonstrate an association between treatment and return to athletic work (51), despite observations that particulate drugs and microparticles accumulate on inflamed synovium near injection sites (52), underscoring that not all biologics translate into performance gains. In the present trial, ligament-associated lameness improved minimally with a relatively flat lameness trajectory to 12 weeks in both eCTM and SOC groups. Although exploratory, ultrasound convergence with performance improvement hypothesized that structural healing may differ or even outpace overt lameness resolution (Supplementary Figure S1).

Joint-specific analyses suggest a potential interaction of pain and performance. SOC-treated joints had transient performance improvement not matched by sustained improvement in joint pain scores. Subsequently, performance declined toward baseline by 26 weeks. ECTM-treated joints demonstrated sustained improvement in joint pain together with continued performance gains through 26 weeks. As hypothesis-generating sub-cohort analyses, eCTM may provide more durable modulation of intra-articular synovial pain responses than some existing biologic approaches. Definitive mechanistic studies would be needed to test this hypothesis.

Multiple-injection protocols for ACS and PRP are widely used in performance horses (53, 54) and occurred in this study, such that SOC had a significantly greater number of re-injections than eCTM due to this practice compared to the single injection of eCTM. In humans, a recent PRP review found no meaningful effect of a single knee injection, and a controlled study suggested that multiple injections may be required for efficacy (up to five in some protocols) (39, 55). In horses, however, three intra-articular ACS injections at two-week intervals did not demonstrate clinical benefit in a controlled study, and a joint infection occurred after a third injection (56). Post-injection infection remains the most frequent serious adverse event reported for PRP and other hemoderivatives in both equine and human literature (55, 56). Because any protocol involving repeated joint injections may increase infection risk (57), the justification for multiple injections should rest on efficacy data or be balanced against options requiring fewer administrations.

### 4.2 Structural remodeling, mechanistic insights, and quantitative validation

Quantitative ultrasound-based TRS data provided a hypothesis that complementary structural evidence of tissue remodeling, particularly in ligament lesions, may coincide with later improvements in performance. Additional studies to test this hypothesis are warranted. The predictive value of early visit ultrasound (2 and 4 weeks) to final ultrasound was high, and, although exploratory, may guide practitioners toward customization of rehabilitation. The data from this study showed that current rehabilitation recommendations were prescriptive for the first 12 weeks, with neither group discriminating the timing of progressive sports medicine practices, such as veterinary ancillary procedures or sports training methods.

TRS remains a novel, not yet fully validated biomarker that, in this study, was used as an exploratory endpoint. Nonetheless, several lines of validation were completed, including repeatability of measurement and significant correlation with clinical lameness. Segmentation reproducibility was high, with a cohort mean Dice coefficient of 0.931 across paired regions of interest, indicating robust agreement between independent passes and above published values for strong intra-rater values (37). Within-horse analyses showed that tendon TRS correlated significantly with limb-level lameness at 12 weeks, a recognized clinical standard, hypothesizing that TRS captured clinically meaningful changes in tissue state. Similarly, the TRS was positively associated with 26-week performance outcome, hypothesizing that early structural remodeling may have a measurable relationship with longer-term functional recovery. These findings do not constitute full validation but provide exploratory hypothesis generation to justify further work to formally validate TRS against clinical and imaging standards in larger cohorts.

Kinematic gait analysis further supported the clinical findings, particularly in forelimb lameness. Forelimb asymmetry indices for impact and push-off improved more in eCTM horses than in SOC horses at several time points, indicating objective improvements in gait symmetry. Hindlimb and therefore combined-limb data were highly variable, potentially for several reasons, including the smaller differences in asymmetry indices recorded for hindlimb in the gait algorithm, possibly related to the lack of head nod combined with the variable situational recording surfaces and conditions among practices. Nonetheless, the Kinematic data suggested, in combination with lameness scores, that eCTM treatment was associated with meaningful functional gains. Additional studies are planned to analyze these gait video data to validate the kinematic platform against standardized lameness and pain scores assigned by experienced clinicians, as has been done for the force plate and other kinematic systems (32–34).

### 4.3 Real-world pragmatic design and subgroup findings

A key feature of this trial is its pragmatic design, which intentionally reflected real-world equine sports medicine practice. Horses were enrolled from eight high-volume performance practices, with a spectrum of joint and soft-tissue conditions, often including multiple lesions within the same limb or bilateral involvement. The SOC arm encompassed the range of biologic treatments currently used by experienced equine clinicians, including hemoderivative products and other injectables, applied according to clinician judgment and manufacturer recommendations. This design enhances external validity and ensures that the comparator represents actual current practice rather than a narrowly defined protocol.

Practice-level enrollment volume and lesion distributions showed the expected variability in case mix between referral practices, but all practices contributed relevant lesion categories, and quasi-randomization within practice mitigated potential confounding by site. Multiple eligible conditions per horse occurred with identical frequency in both groups, and mixed-effects models that accounted for within-horse clustering yielded treatment effects consistent with the primary analysis.

Inevitably, pragmatic designs introduce heterogeneity in lesion types, practice patterns, and ancillary therapies. Subgroup analyses and sensitivity tests were therefore undertaken to explore whether the main treatment effect was consistent across key strata while acknowledging that these analyses were not prespecified and some are underpowered. Exploratory comparisons were in alignment with the primary outcome results, with directional consistency favoring eCTM over SOC broadly across joints and hemoderivative product-class analyses, both of which included practices with varying case mixes and volumes. Product-class analyses suggested that the effectiveness of eCTM over SOC seen in primary outcomes was preserved when SOC was restricted to autologous hemoderivative products. Exploratory analyses specifically hypothesized that autologous APS preserved the findings of the primary outcomes, favoring eCTM with a clinical advantage over APS for most outcomes, despite the small sample size and *post hoc* nature of this comparison. Despite technological advances in the generation of hemoderivatives, these findings do not dispel the concern that donor variability and differences among autologous systems may contribute to elusive repeatable clinical responses in practice (11–15).

In addition to autologous products, several non-umbilical equine tissue allografts have been investigated, particularly liquid amnion-based preparations and allogeneic allogeneic dental pulp–derived tissues (34, 38, 58) Liquid amnion allografts have shown acceptable safety in ligament injury in uncontrolled clinical case studies, but controlled trials are still lacking (38). By contrast, equine dental pulp connective tissue particles have been evaluated in a prospective randomized, blinded, placebo-controlled study of naturally occurring osteoarthritis, desmitis, and tendonitis, and produced significant short-term improvements in lameness and comfort compared with controls (34), highlighting the potential of standardized allogeneic grafts to provide effective off-the-shelf musculoskeletal therapies.

The smaller subgroup analyses must be interpreted cautiously, but are associated with similar trajectories as the primary outcomes. The consistency of the treatment effect in sensitivity analyses, including models that accounted for within-horse clustering, supports the association of eCTM treatment improvements in the primary clinical outcomes.

### 4.4 Safety and owner-reported outcomes

Safety is a central consideration when introducing a new ortho-biologic therapy. In this study, eCTM was well tolerated. Product-related adverse events were infrequent, self-limited, and occurred early after injection. SOC-treatments were associated with a higher frequency of product-related adverse events overall and in joint conditions specifically, including a small number of serious events. The overall reports were low in both arms of the study and provide a favorable safety profile for eCTM in these musculoskeletal tissues.

Adverse reactions from intra-articular autologous hemoderivatives are generally considered uncommon (<5%), yet surveys indicate that 56, 59, 42, and 20% of equine specialists have observed negative effects after MSCs, PRP, ACS, and APS, respectively, most often local inflammation or infection (59–61). Evidence-based, controlled joint-safety data in horses remain limited, and in a human-controlled arthritis trial, PRP caused significantly more adverse events—predominantly joint flares—than plasma or saline (62), with PRP-associated flares widely reported in human literature (56, 62). Equine reports of joint flares with ACS, APS, cell therapies, and polyacrylamide rely largely on retrospective surveys, in which 10–18.2% of veterinarians reported >5% reaction rates; although 4.0% polyacrylamide appeared safe in normal equine joints (63), practice-based surveys identified acute joint pain in TB racehorses as a contraindication and noted that 18.1% of veterinarians observed >5% joint reaction rates (61). In the present trial, two serious joint adverse reactions occurred after polyacrylamide, and one persisted at subsequent follow-up evaluations.

Classification of adverse events is partly subjective, and site investigators may sometimes attribute complications to management rather than to the injected product. For this study, a central review that objectively followed the definitions in the protocol judged several cases as plausibly product-related in both groups. Reclassification as possibly product-related events in sensitivity analyses did not change the statistical significance of the safety profile that favored eCTM. The data suggested that eCTM had a favorable safety profile.

Owner-reported outcomes provide an additional perspective on treatment impact. Client assessments indicated significant improvements in health and quality-of-life indices over time in both groups, consistent with the clinical trajectories. However, at 26 weeks, owners of eCTM-treated horses reported higher scores for body condition, coat quality, overall health, and composite quality of life than owners of SOC horses, suggesting the product will be well received by owners and possibly reflect subclinical soft measures of health. Scores for demeanor, appetite, comfort at turnout, and in the stall improved in both groups, which may reflect the combined effect of time, treatment, rehabilitation, and management changes.

### 4.5 Limitations

This pragmatic clinical study was open-label and unblinded within practices. Blinding of veterinarians and owners was not feasible because SOC biologic treatments required visible blood collection and preparation, and it would not have been acceptable in routine performance-horse practice to have injections administered by a separate masked operator using undisclosed products. Lack of blinding can introduce expectation and observer bias. Several safeguards were implemented to protect internal validity, including quasi-randomized allocation within practices, prespecified eligibility criteria and outcome definitions, consensus training and standardized scoring for project veterinarians, use of objective TRS and kinematic measures in subsets, sequential case allocation, and equivalent client management and non-contingent compensation for veterinarians and clients. The consistency of the treatment effect across intention-to-treat and per-protocol analyses, and in sensitivity analyses accounting for multiple conditions per horse and group-level and practice-level deviations, supports the robustness of the primary findings despite the open-label design.

The pragmatic practice-based design introduced heterogeneity in lesion types and product choices within the SOC arm, which may have contributed to variability in outcomes. While this mirrors real-world practice and enhances external validity, the variability must be compensated for by inclusion of sufficient case numbers and can complicate attribution of effects to specific pathologies (conditions) or subgroups of comparator products. The trial was powered for the overall comparison between eCTM and SOC in the pooled musculoskeletal population; subgroup analyses by lesion type, specific control products, or practice would be mostly hypothesis-generating.

Some horses contributed more than one treated condition, and some practices varied in the intended allocation balance; however, the intended balance was maintained centrally without intervention. Mixed-effects models with random effects for horse and condition were used to account for within-horse clustering and sensitivity analyses. Excluding practice(s) with low SOC assignment had no measurable impact on the magnitude of the treatment effect in the primary analysis. Nonetheless, residual imbalance and unmeasured practice-level factors cannot be completely ruled out as contributing to variability.

The quantitative ultrasound-based TRS used in this study is a novel metric and, although included as an exploratory endpoint, has not yet undergone full formal validation. TRS findings are therefore interpreted as hypothesis-generating, and larger dedicated prospective (predictive) validation studies against established imaging and clinical standards are the next steps.

While the integration of TRS and kinematic data adds valuable mechanistic insight, these analyses were limited to subsets of horses with high-quality imaging or motion data and should be considered supportive of primary outcomes. The tendon TRS subgroup was underpowered for independent conclusions.

Finally, long term 26-week endpoint was considered an optimal interval that would determine whether horses had resumed or were expected to resume performance, capture early returns to work, and maintain complete follow-up; recognition that after 26 weeks, competing factors in competitive performance horses, such as new injuries, sales, changes in management and trainers, limited performance lifespan unrelated to the project, and loss of client contact could erode data quality. Longer-term follow-up, due to data loss, may not be sufficiently powered to contribute statistically to controlled effectiveness computations. Longer-term follow-up may be valuable retrospectively for both controls and eCTM, but it is beyond the scope of this highly regulated and controlled project.

### 4.6 Clinical implications and future directions

This prospective and contemporaneously controlled study provides clinically relevant evidence that cryopreserved umbilical allografts improve outcomes for performance horses with musculoskeletal lameness compared with standard treatments currently used in practice. The combination of earlier and greater improvements in lameness, pain, and swelling, sustained performance gains through 26 weeks, a favorable safety profile, supportive structural and kinematic data, along with consistent composition, lack of patient-side processing, and as a single injection suggests that eCTM may offer a therapeutic option available for joint and soft-tissue conditions in performance horses. This study specifically supports consideration of eCTM in horses with performance-limiting lameness where previous biologic treatments have provided only transient benefit, as for the horses in this study. Future studies in stratified cohorts, optimal dosing, and re-treatment strategies are planned. Additional mechanistic work integrating imaging, biomarker, and clinical data may further clarify how eCTM influences tissue remodeling and how best to select candidates most likely to benefit.

This multi-centered prospective quasi-randomized open-label controlled clinical trial with long-term follow-up on over a hundred of horses in real-world practice conditions serves to fill a gap in knowledge in the field of biologics. Fully randomized and blinded prospective controlled clinical trials (RCTs), as defined by CONSORT guidelines have not been done in horses and may or may not may provide complementary efficacy data, but there are also disadvantages to RCTs. RCTs are highly controlled with fewer preselected animals, thus limiting the generalizability of the results to the broader population of patients with the condition being represented. RCTs also tend to study treatments in idealized environments, which may not be perfectly in line with real-world usage of the treatment, possibly negating the clinical efficacy when practiced in the real world. Overall, reports of equine clinical trials on lameness are scarce, particularly for biologics, and additional studies are needed.

## 5 Summary and conclusion

In this pragmatic setting, a single off-the-shelf injection of eCTM was associated with earlier, greater, and more durable improvements in clinical and performance outcomes than clinician-selected non-steroidal SOC. Overall, these positive findings suggest eCTM may represent a consistent, single-dose alternative to clinician SOC approaches for selected musculoskeletal conditions in performance horses.

## Data Availability

The original contributions presented in the study are included in the article/supplementary material, further inquiries can be directed to the corresponding author/s.

## Supplementary material

SUPPLEMENTARY FIGURE S1 Representative cases of proximal suspensory injury from the standard-of-care (SOC) control (left; cross section images) showing progressive structural deterioration over 12 weeks (TRS score of 31) and equicenta^®^ CTM (eCTM) (right; longitudinal images) showing an early macrostructural (MS core response and late-emerging field remodeling response) (quality layer) contributing to a responding TRS of 61. Trajectory charts display these scores over time. B-mode ultrasound (top rows) shows transverse heterogeneous ligament cross/long-sections. The Ocean Floor topographic map (middle row; teal = healthy tissue elevation, dark blue/navy = lesion depth) shows heterogeneous regions of surrounding tissue quality layers. The 3D Terrain rendering (bottom rows) provides a volumetric perspective of the same data, with surface peaks (green/teal) representing healthy tissue architecture and valleys (pink/red) indicating regions of reduced tissue quality. At baselines, the terrain showed a mixed landscape with identifiable peaks and a central depression in both cases. The terrain map provides an intuitive representation of treatment response: healthy tissue appears as elevated peaks, while lesion regions manifest as valleys or craters. Both cases use identical rendering parameters (camera angle, color mapping, elevation scaling) to enable direct visual comparison between SOC control and eCTM outcomes. Left Control Interpretation: By week 13, the cross-section B-mode image showed more diffuse loss of echogenicity. The Ocean Floor map revealed uniform deepening across the entire ligament, with expansion of the dark navy lesion zone. The 3D Terrain rendering made the deterioration apparent: the surface collapsed to a predominantly flat, pink/red plane with minimal residual peaks, indicating near-complete loss of organized tissue architecture. The contrast between the baseline and week 13 terrain maps provided an intuitive visual summary of the progressive structural deterioration. The corresponding trajectory chart demonstrated that TRS declined from 50 (Baseline) to 51 (week 2) to 44 (week 4, crossing below the Not Responding threshold) to 31 at week 13. MS Core collapsed to 13, indicating marked loss of structural scaffold. Quality Layer declined more gradually to 42 and suggested that surface-level tissue properties were partially preserved despite deep structural failure. Equicenta^®^ CTM Interpretation: Representative case from the equicenta® CTM group demonstrated an early MS core response and late-emerging field remodeling response in a proximal suspensory ligament (PSL), imaged in the longitudinal plane. By week 13, the B-mode image showed improved fiber organization and more uniform echogenicity. The Ocean Floor map demonstrated shallower, more uniform teal coloration with less dark blue, indicating tissue quality improvement. The 3D Terrain rendering revealed marked terrain building: peaks have grown taller and more numerous (green/yellow), the central valley (more pink, less red) has filled in, and the overall surface elevation had increased substantially. This terrain transformation—from low, rough ground to an elevated, peaked landscape—provides an intuitive visual representation of the structural remodeling. The corresponding trajectory chart demonstrated that TRS rose early from 50 (baseline) to responding scores by week 2. The primary contributor to TRS score response was MS Core that had a robust early response, peaking by week 4. Quality Layer lagged, declining slightly in field remodeling before recovery. This dissociation between MS Core and Quality Layer is characteristic of a late-responding pattern in which deep structural reorganization precedes surface-level tissue maturation.

SUPPLEMENTARY FIGURE S2 Plot of the positive equicenta^®^ CTM (eCTM) effect for the ultrasound tissue response score (TRS) as compared to SOC alongside the plot of the positive eCTM effect for performance compared to SOC showing convergence and overlap most pronounced in ligaments with a cohen’s d score of 0.96 reflecting high clinical relevance.

SUPPLEMENTARY FIGURE S3 Subgroup analysis comparing autologous protein solution (APS) to equicenta^®^ CTM (eCTM): Cumulative clinical improvement trajectories from baseline score (mean ± SEM) for the outcomes of clinical lameness, pain, swelling over 12 weeks for equicenta^®^ CTM or APS standard of care (SOC) favors eCTM. Greater variability and lower numbers in this post-hoc subgroup is interpreted with caution but suggests that eCTM outperformed APS in this real-world clinical setting of lame performance horses.

## Glossary

AAEP: American Association of Equine Practitioners
ACS: Autologous conditioned serum
APS: Autologous protein solution
AVMA: American Veterinary Medical Association
CBC: Complete blood count
CI: Confidence interval
CTM: Connective tissue matrix
CV: Coefficient of variation
eCTM: equicenta® CTM
ETCR: East Tennessee Clinical Research
FEI: Federation Equestra Internationale
GAG: Glycosaminoglycan
HA: Hyaluronan
MSC: Mesenchymal stromal cell
NSAID: Non-steroidal anti-inflammatory drug
OA: Osteoarthritis
OR: Odds ratio
PR: Person responsible
PRP: Platelet rich plasma
PV: Project veterinarian
ROI: Region(s) of interest
SEM: Standard error of the mean
SI: Site investigator
SOC: Standard of care
ST: Soft tissue (ligament and tendon)
TB: Thoroughbred
TRS: Tissue remodeling score
VCT: Veterinary clinical trial
VPO: Veterinary Performance Outcome
