# Supplementary material for: Cryopreserved umbilical allograft (equicenta® CTM) improves clinical outcomes compared with clinician-selected standard of care in performance horses: a pragmatic prospective controlled trial
Source: Front Vet Sci. 2026 Aug 4;13:1833933. doi: 10.3389/fvets.2026.1833933 (PMC13483253; doi:10.3389/fvets.2026.1833933)
Supplement: Supplementary file 1 [file Table_1.docx]

**SUPPLEMENTARY TABLE S1.** Body weight of horses that completed the clinical trial (kg)

|  | SOC Control | equicenta® CTM | Overall |
| --- | --- | --- | --- |
| Week 0 | 496 ± 8.1 | 487 ± 5.0 | 492 ± 4.2 |
| Week 12 | 513 ± 7.0 | 492 ± 4.4 | 503 ± 3.8 |
| **Mean by group** | 504.5 | 489.5 |  |

SOC Control and equicenta® CTM did not statistically differ
