# Supplementary material for: Cryopreserved umbilical allograft (equicenta® CTM) improves clinical outcomes compared with clinician-selected standard of care in performance horses: a pragmatic prospective controlled trial
Source: Front Vet Sci. 2026 Aug 4;13:1833933. doi: 10.3389/fvets.2026.1833933 (PMC13483253; doi:10.3389/fvets.2026.1833933)
Supplement: Supplementary file 2 [file Table_2.docx]

**SUPPLEMENTAL TABLE S2.** Mean body condition score per horse recorded by veterinarians for SOC control or equicenta® CTM expressed as counts and percent for weeks 0-12 of the clinical trial.

|  | SOC Control | | equicenta® CTM | |
| --- | --- | --- | --- | --- |
| Body Score | *n* | % | *n* | % |
| 3 | 1 | 2.1 | 0 | 0.0 |
| 4 | 7 | 14.9 | 7 | 7.7 |
| 5 | 23 | 48.9 | 61 | 67.0 |
| 6 | 10 | 21.3 | 18 | 19.8 |
| 7 | 6 | 12.8 | 4 | 4.4 |
| 8 | 0 | 0.0 | 1 | 1.1 |
| **Total** | 47 |  | 91 |  |

SOC= standard of care; Mean body scores remained within the protocol range (3-8) for SOC and equicenta® CTM. Both equicenta®CTM and SOC had 5 as the most common body score.
