# Supplementary material for: Cryopreserved umbilical allograft (equicenta® CTM) improves clinical outcomes compared with clinician-selected standard of care in performance horses: a pragmatic prospective controlled trial
Source: Front Vet Sci. 2026 Aug 4;13:1833933. doi: 10.3389/fvets.2026.1833933 (PMC13483253; doi:10.3389/fvets.2026.1833933)
Supplement: Supplementary file 3 [file Table_3.docx]

**SUPPLEMENTARY TABLE S3.** Distribution of practice by recruitment map of horses and musculoskeletal conditions enrolled in equicenta® CTM (eCTM) or standard of care (SOC) (n = 138 horses, 195 conditions).

| Region | Horses *n* (%) | Conditions *n* (%) | Joint *n* (%) | Ligament *n* (%) | Tendon *n* (%) |
| --- | --- | --- | --- | --- | --- |
| **Kentucky** | 37 (26.8) | 53 (27.2) | 40 (75.5) | 10 (18.9) | 3 (5.7) |
| SOC | 11 (29.7) | 17 (32.1) | 16 (94.1) | 1 (5.9) | 0 (0.0) |
| eCTM | 26 (70.3) | 36 (67.9) | 24 (66.7) | 9 (25.0) | 3 (8.3) |
| **North Carolina** | 35 (25.4) | 44 (22.6) | 23 (52.3) | 15 (34.1) | 6 (13.6) |
| SOC | 18 (51.4) | 21 (47.7) | 11 (52.4) | 8 (38.1) | 2 (9.5) |
| eCTM | 17 (48.6) | 23 (52.3) | 12 (52.2) | 7 (30.4) | 4 (17.4) |
| **Ohio** | 14 (10.1) | 21 (10.8) | 14 (66.7) | 2 (9.5) | 5 (23.8) |
| SOC | 6 (42.9) | 9 (42.9) | 6 (66.7) | 1 (11.1) | 2 (22.2) |
| eCTM | 8 (57.1) | 12 (57.1) | 8 (66.7) | 1 (8.3) | 3 (25.0) |
| **Texas** | 10 (7.2) | 17 (8.7) | 15 (88.2) | 0 (0.0) | 2 (11.8) |
| SOC | 5 (50.0) | 10 (58.8) | 10 (100.0) | 0 (0.0) | 0 (0.0) |
| eCTM | 5 (50.0) | 7 (41.2) | 5 (71.4) | 0 (0.0) | 2 (28.6) |
| **Florida** | 42 (30.4) | 60 (30.8) | 12 (20.0) | 36 (60.0) | 12 (20.0) |
| SOC | 7 (16.7) | 10 (16.7) | 3 (30.0) | 5 (50.0) | 2 (20.0) |
| eCTM | 35 (83.3) | 50 (83.3) | 9 (18.0) | 31 (62.0) | 10 (20.0) |
| **Total** | **138 (100.0)** | **195 (100.0)** | **104 (53.3)** | **63 (32.3)** | **28 (14.4)** |
| SOC | 47 (34.1) | 67 (34.4) | 46 (68.7) | 15 (22.4) | 6 (9.0) |
| eCTM | 91 (65.9) | 128 (65.6) | 58 (45.3) | 48 (37.5) | 22 (17.2) |

Ranked in order of most to least number of horses per Project Veterinarian at the practices in the clinical study.
