# Supplementary material for: Cryopreserved umbilical allograft (equicenta® CTM) improves clinical outcomes compared with clinician-selected standard of care in performance horses: a pragmatic prospective controlled trial
Source: Front Vet Sci. 2026 Aug 4;13:1833933. doi: 10.3389/fvets.2026.1833933 (PMC13483253; doi:10.3389/fvets.2026.1833933)
Supplement: Supplementary file 4 [file Table_4.docx]

**SUPPLEMENTARY TABLE S4. Distribution of primary conditions per horse assigned to standard of care control (SOC) or equicenta^®^ CTM**

|  | SOC (n = 47) | | eCTM (n = 91) | |
| --- | --- | --- | --- | --- |
|  | n | % | n | % |
| **A. Primary Conditions*** |  |  |  |  |
| 1 condition (single site). | 30 | 63.8% | 58 | 63.7% |
| 2 conditions in one lame limb. | 1 | 2.1% | 11 | 12.1% |
| 1 condition in two lame limbs | 14 | 29.8% | 19 | 20.9% |
| 3 conditions | 2 | 4.2% | 3 | 3.3% |
| **Total horses with >1 condition** | 17 | 36.2% | 33 | 36.3% |
| **Total horses** | 47 | 100.0% | 91 | 100.0% |

*There was no significant difference between groups in distribution of primary conditions.
