# Supplementary material for: Cryopreserved umbilical allograft (equicenta® CTM) improves clinical outcomes compared with clinician-selected standard of care in performance horses: a pragmatic prospective controlled trial
Source: Front Vet Sci. 2026 Aug 4;13:1833933. doi: 10.3389/fvets.2026.1833933 (PMC13483253; doi:10.3389/fvets.2026.1833933)
Supplement: Supplementary file 5 [file Table_5.docx]

**SUPPLEMENTARY TABLE S5.** Details of adverse events by horse and by report within the population of all injections for equicenta® CTM (eCTM) or Standard of Care (SOC).

|  | **Severity** | **Report #** | **Horse #** | **Practice** | **Products** | **Breeds** | **Discipline** | **Diagnosis** | **Signs** | **Wk 12 Lameness** | **Wk 26 VPO** |
| --- | --- | --- | --- | --- | --- | --- | --- | --- | --- | --- | --- |
| **Veterinarian Scores** | Serious / SOC | 2 | 1 | P2 | PAAG | WB | Jumper | LH/RH: Bilateral hind fetlock arthritis | Lameness Swelling | 3 | euthanized |
|  | Nonserious / SOC | 4 | 1 | P6 | PAAG | TB | Hunter | RH: tarsocrural arthritis | Marked effusion Lameness | 3 | 2 |
|  | Serious / eCTM | 0 | 0 | none | none | none | none | none | none | none | none |
|  | Nonserious / eCTM | 3 | 3 | P1  P3  P8 | eCTM  eCTM  eCTM | Hackey  STDB  QH | Driving  Racing  Racing | LH: Stifle arthritis  LF: SDF tendonitis  RF: DDF tendonitis (insertion) | Lame Swelling Lameness | 0  0  4 | 4  3  2 |
| **Total Veterinarian Scores** | ***SOC*** | **6** | **2** |  |  |  |  |  |  |  |  |
|  | ***eCTM*** | **3*** | **3** |  |  |  |  |  |  |  |  |
| **Central Review** | Serious SOC | 9 | 4† | P2  P6  P6  P2 | Fat MSC  ACS  PAAG  PAAG | QH  WB  TB  WB | Western  Hunter  Hunter  Jumper | LH: Chronic active PSD  RF: Coffin joint arthritis  RH: tarsocrural arthritis  LH/RH: Fetlock arthritis | Lame Swollen Painful | 1  0  3  3 | 3  4  2  euthanized |
|  | Nonserious SOC | 4 | 4 | P2  P2  P7   P6 | APS  APS  PRP   PAAG | QH  WB  WB   TB | Western  Eventing  Hunter   Hunter | LH: Chronic active PSD  RF: PSD desmopathy  LH/RH: Suspensory branch desmitis  RH: tarsocrural arthritis | Lame  Lame  Swelling   Effusion | 1  2  0   3 | 3  2  1   2 |
|  | Serious eCTM | 1 | 1 | P2 | eCTM | QH | Western | RH: tarsocrural arthritis | Lame Effusion | 3 | 2 |
|  | Nonserious eCTM | 4 | 4† | P2  P1  P3  P8 | eCTM  eCTM  eCTM  eCTM | TB  Hackey  STDB  QH | Eventing  Driving  Racing  Racing | RH: LSB desmopathy  LH: Stifle arthritis  LF: SDF tendonitis  RF: DDF tendonitis (insertion) | Swelling  Lame  Swelling  Lameness | 4  0  0  4 | 2  4  3  2 |
| **Total Central Review** | ***SOC*** | **13** | **8** |  |  |  |  |  |  |  |  |
|  | ***eCTM*** | **5*** | **5**** |  |  |  |  |  |  |  |  |

*Reported Adverse Reactions by the veterinarian (p<0.04) and central review (p<0.002) were less frequent in eCTM than eSOC.

** After central review, fewer horses had an adverse reaction reported in eCTM than SOC (p<0.04)

† One horse was a rescue injection

PAAG=polyacrylamide gel, WB=Warmblood, TB=Thoroughbred, QH = Quarter Horse, STDB=Standardbred, MSC=mesenchymal stromal cell, ACS=autologous conditioned serum, PRP=platelet-rich plasma

DDF=deep flexor tendon; SDF=superficial flexor tendon; LSB=lateral suspensory branch; PSD=proximal suspensory desmitis
