# Supplementary material for: Cryopreserved umbilical allograft (equicenta® CTM) improves clinical outcomes compared with clinician-selected standard of care in performance horses: a pragmatic prospective controlled trial
Source: Front Vet Sci. 2026 Aug 4;13:1833933. doi: 10.3389/fvets.2026.1833933 (PMC13483253; doi:10.3389/fvets.2026.1833933)
Supplement: Supplementary file 6 [file Table_6.docx]

**SUPPLEMENTARY TABLE S6.** Details for horses that received rescue injections as per protocol at the target site for standard of care (SOC) and equicenta^®^ CTM (eCTM).

| **Rescue Injections** | **Lameness at Rescue** | **Limb** | **Diagnosis** | **Product** |  |  |
| --- | --- | --- | --- | --- | --- | --- |
| **Rescue — SOC→SOC** | | | | | | |
|  | 3 | RH | Tarsocrural Arthritis | ACS to PAAG |  |  |
|  | 2 | LF | SDF Tendonitis. | APS to Fat MSC |  |  |
| **Rescue —SOC → eCTM — crossover** | | | | | | |
|  | 4 | RH | Hip Arthritis | PRP |  |  |
|  | 3 | LH | Suspensory Desmitis | HA and PSGAG |  |  |
|  | 2 | LH | Suspensory Desmitis | Fat MSC |  |  |
|  | 3 | LH | Stifle Arthritis | PRP |  |  |
|  | 1 | LH | Fetlock Arthritis | HA |  |  |
|  | 1 | LH | Fetlock Arthritis | PAAG |  |  |
|  | 2 | RF | LSB Desmitis | PSGAG |  |  |
|  | 2 | LF | Suspensory Desmitis. | PRP |  |  |
|  | 3 | LH | Stifle Arthritis | Cultured Bone  marrow MSC |  |  |
|  | 1 | LH | Stifle Arthritis | PPP |  |  |
|  | 1 | RH | Stifle Arthritis | PPP |  |  |
| **Rescue —eCTM → eCTM** | | | | | | |
|  | 3 | LH | Collateral Ligament Desmitis | eCTM |  |  |
|  | 3 | LH | Tarsocrural Synovitis | eCTM |  |  |
|  | 4 | RH | Hip Arthritis | eCTM |  |  |
|  | 2 | RF | Coffin Joint Arthritis | eCTM |  |  |
|  | 3 | RH | LSB Desmitis | eCTM |  |  |
|  | 3 | LF | Coffin Joint Arthritis (cartilage damage on MRI) | eCTM |  |  |
|  | 3 | RH | Tarsocrural Arthritis | eCTM |  |  |
|  | 4 | LF | Carpus Arthritis | eCTM |  |  |
|  | 2 | RF | Carpus Arthritis | eCTM |  |  |

LSB = Lateral suspensory branch; ACS = Autologous Conditioned Serum; PAAG = polyacrylamide gel; APS = Autologous protein solution; PRP = Platelet-rich plasma; HA = Hyaluronic acid; PSGAG= polysulfated glycosaminoglycan; PPP= platelet-poor plasma; MSC=mesenchymal stromal cells;

The odds of hindlimbs (H) having rescue injections compared to forelimbs was 4.6:1 (p<0.02); The odds of SOC having more hindlimbs rescued than eCTM was 2.6:1 but did not statistically differ (p>0.2).
